# Effects of Mosquito Biology on Modeled Chikungunya Virus Invasion Potential in Florida

**DOI:** 10.3390/v12080830

**Published:** 2020-07-30

**Authors:** Cynthia C. Lord, L. Philip Lounibos, Joseph J. Pohedra, Barry W. Alto

**Affiliations:** Florida Medical Entomology Laboratory, University of Florida—IFAS, 200 9th St. SE, Vero Beach, FL 32962, USA; lounibos@ufl.edu (L.P.L.); jpohedra@yahoo.com (J.J.P.); bwalto@ufl.edu (B.W.A.)

**Keywords:** Chikungunya virus, invasion, transmission dynamics, *Aedes aegypti*, Aedes albopictus, mathematical model

## Abstract

Arboviruses transmitted by *Aedes aegypti* and *Aedes albopictus* have been introduced to Florida on many occasions. Infrequently, these introductions lead to sporadic local transmission and, more rarely, sustained local transmission. Both mosquito species are present in Florida, with spatio-temporal variation in population composition. We developed a two-vector compartmental, deterministic model to investigate factors influencing Chikungunya virus (CHIKV) establishment. The model includes a nonlinear, temperature-dependent mosquito mortality function based on minimum mortality in a central temperature region. Latin Hypercube sampling was used to generate parameter sets used to simulate transmission dynamics, following the introduction of one infected human. The analysis was repeated for three values of the mortality function central temperature. Mean annual temperature was consistently important in the likelihood of epidemics, and epidemics increased as the central temperature increased. *Ae. albopictus* recruitment was influential at the lowest central temperature while *Ae. aegypti* recruitment was influential at higher central temperatures. Our results indicate that the likelihood of CHIKV establishment may vary, but overall Florida is permissive for introductions. Model outcomes were sensitive to the specifics of mosquito mortality. Mosquito biology parameters are variable, and improved understanding of this variation will improve our ability to predict the outcome of introductions.

## 1. Introduction

Chikungunya virus (CHIKV) is an *Alphavirus* in the family Togiviridae, native to Africa, which causes human disease and is transmitted by *Aedes* mosquitoes, primarily invasive *Stegomyia*. Zoonotic cycles between sylvatic *Aedes* species and non-human primates occur in Africa [1], with spillover to the human population, but elsewhere the transmission cycle is primarily between *Ae. aegypti* or *Ae. albopictus* (in its invasive range) and humans. *Aedes aegypti* is considered the most common vector, but outbreaks have occurred where *Aedes albopictus* is the dominant vector, such as in La Reúnion in 2005–2006, India (2006), and Italy (2007) [1,2]. Three major lineages are recognized (East, Central, and South African (ECSA), Asian, and West African (WA)), with an additional sublineage of ECSA (Indian Ocean lineage, IOL). The genotypes of these lineages have diverged, and virulence in humans varies among lineages [3]. In addition, a mutation in the envelope protein (E1-A226V) that enhances transmission and reduces the extrinsic incubation period (EIP) in *Ae. albopictus* was found in the ECSA lineage [4], but the lineage is heterogeneous for the presence of the mutation. Globally, CHIKV transmission is ongoing [5] but can increase to outbreak levels and invade new areas. In 2014, there were high levels of CHIKV transmission in the Americas and the Caribbean, leading to concern about introduction of the virus to new areas. The risk of introductions and outbreaks continues, as illustrated by the ongoing outbreak in northeastern Brazil [6], with over 37,000 cases to date. This outbreak consists of ECSA lineage CHIKV, without the mutation enhancing transmission in *Ae. albopictus* [7].

The risk of arbovirus introduction in Florida is high due to a permissive environment, the presence of known vectors, and high movement of hosts, especially travelers, into the state from endemic or outbreak regions. In 2010, a local outbreak of dengue virus (DENV) occurred in Key West, FL, USA [8], increasing concern about local transmission of exotic arboviruses, especially those transmitted by *Ae. aegypti* and *Ae. albopictus,* which are the only mosquitoes in the subgenus *Stegomyia* currently present in Florida. Subsequently, many imported cases of dengue have been reported [9]. There have also been sporadic cases with no travel history and of different serotypes, suggested repeated local transmission, although without sustained transmission leading to outbreaks [10,11]. Concurrent with high levels of CHIKV transmission elsewhere, in 2014 there were multiple introduced cases of CHIKV in Florida [12], showing the invasion potential. During this time, low levels of local transmission occurred, with one cluster in southeast FL [13]. Further illustrating the potential for arbovirus invasion of Florida, in 2015–2016 there was widespread transmission of Zika virus (ZIKV) in South and Central America and the Caribbean [14]. The introduction pressure was high, with >300 introduced cases in FL [15]. Subsequently, autochthonous transmission occurred in multiple locations in Florida [15]. Currently, the factors leading to successful invasion and autochthonous transmission of arboviruses are not well known.

Modeling has been used extensively to study the transmission dynamics of mosquito borne arboviruses, across many systems and utilizing varied methodology (e.g., [16,17,18,19,20] also see reviews in [21,22,23,24]). Temperature has been shown to be a key element, influencing many aspects of mosquito and virus dynamics, but with a relatively wide range permitting transmission [25]. Previous models of mosquito transmitted arboviruses have included varying levels of detail in mosquito population dynamics and biology, genotype differences within viruses, host dynamics, and other complexities. Manore et al. [20] modeled DENV and CHIKV in *Ae. albopictus* and *Ae. aegypti,* concluding that reductions in the biting rate and vector to host ratio would have the most impact on initial epidemics and endemic equilibria for all combinations considered. They compared the Asian lineage of CHIKV in *Ae. aegypti* (their notation CHIKV-A) with the ECSA lineage in *Ae. albopictus,* including the transmission-enhancing mutation (their notation CHIKV-R). Most parameters varied between the two combinations, with CHIKV-R having wider ranges or higher transmission rates, a shorter EIP, and lower turnover of the mosquito population. Under their assumptions and parameter estimates, the CHIKV-R combination had higher prevalence and faster epidemics than did the CHIKV-A combination. Their analysis suggests that the CHIKV-R combination would require integrated, larger scale mitigation strategies than the CHIKV-A combination to achieve a similar reduction in epidemic peaks. They did not consider the situation with a mixed mosquito population (both vectors present simultaneously) nor the potential variation in transmission efficiency with different combinations of CHIKV genetics and mosquito species. Both of these situations could occur with introductions of CHIKV into Florida, and may affect the likelihood of a successful invasion (establishment and local transmission).

Many arboviral epidemiological models have used simplified mosquito mortality functions, such as a fixed rate (e.g., [20]) or increasing with temperature (e.g., [17,18,19]). Adult mosquito mortality in the field is complex, and is likely influenced by many factors including climate, age, predators, and the larval environment. Some of these factors have been investigated in some detail (e.g., age [26]; temperature [27]) but an improved understanding of how complexity in mosquito mortality affects arboviral epidemiology is needed. In particular, increasing mortality with temperature may be appropriate in some situations, but mortality may also increase with cooler temperatures. One example is the complex polynomial fit by Yang et al. [27] to laboratory mortality data for *Ae. aegypti*. However, it is unknown whether this function is applicable to other populations or species or in the field, and the parameters are difficult to interpret biologically. As mosquito mortality directly impacts the probability of survival through the extrinsic incubation period, considering other complex, biologically relevant relationships is important in addressing the risk of invasion in diverse locations.

The distribution of *Ae. aegypti* and *Ae. albopictus* across Florida is highly variable in both space and time [28,29,30,31,32]. The two species share many characteristics, including larval development in a variety of small water-holding containers such as discarded tires or plant pots. However, they also differ in aspects important to arbovirus transmission, such as host preference [33,34,35]. The domesticated form of *Ae. aegypti* prefers to feed on humans (e.g., [33,34]) while *Ae. albopictus* is more opportunistic, but will readily feed on humans (e.g., [35,36,37]). Both are competent vectors for DENV, Zika virus (ZIKV), and CHIKV, although there are population and strain differences in virus–mosquito interactions. With this background, the risk of establishment and local transmission in Florida is expected to be highly variable, and a better understanding of the factors affecting this risk is critically needed. Process-based epidemiological modeling is an effective way to consider many combinations of factors to assess their contribution to outcomes such as local transmission and number of infected people. A two-vector WNV model for Florida demonstrated that the presence of two vector species can influence West Nile virus dynamics [19]. However, that study focused on species with different seasonal patterns but similar biology and transmission, while *Aedes*-transmitted arboviruses in Florida are likely to be more similar in seasonal patterns but have differences in biology and transmission. Given the variation in the major CHIKV vectors in Florida, understanding potential invasions requires inclusion of both vectors and varying relative abundance in models to assess how these factors influence arboviral dynamics.

To address questions about the likelihood of successful establishment of CHIKV into Florida against a background of fluctuating populations of two mosquito species, we adapted a previous two-vector arbovirus model [19]. This model allows different population sizes, both absolute and relative, of *Ae. aegypti* and *Ae. albopictus*, CHIKV strain variation, and includes a nonlinear relationship between adult mosquito mortality and temperature. We used the model to investigate factors influencing risk of invasion of CHIKV into different locations in Florida, and to consider the impact of fixed parameters on model outcomes.

## 2. Materials and Methods

### 2.1. Model Structure

**Figure 1:**
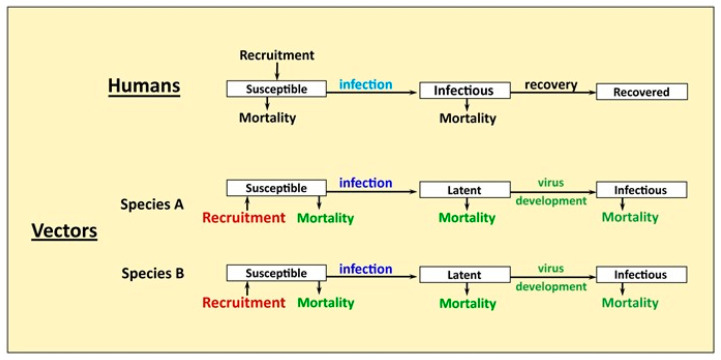
Flow chart of model. Temperature dependent functions shown in green text, seasonal mosquito recruitment in red, and strain-specific transmission parameters (infection) in light blue (humans) and dark blue (mosquitoes). Species A is *Aedes aegpti* and Species B is *Aedes albopictus*.

The basic model structure follows an earlier deterministic two-vector, multiple host arbovirus model [19], which was developed from one-vector, two-host arbovirus models [17,18]. Two vectors were retained but the host structure was simplified to consider only one host type, as there are no known alternate hosts for CHIKV in Florida (Figure 1). The model is SIR (Susceptible–Infectious–Recovered) for hosts (humans) and SEI (Susceptible–Exposed or latent–Infectious) for vectors. Virus genotype differences are included by altering relevant parameters. Below, we detail the changes made to the structure, equations, and parameters of the model to consider CHIKV introductions into Florida. Other model structure and equations can be found in the references above. The model has a time step of 1 day and is run for 730 days following virus introduction. A combination of stratified random sampling and systematic variation was used for sensitivity analysis, detailed below. Model parameters and ranges used in the sensitivity analysis are given in Table 1.

#### 2.1.1. Temperature Cycle

The previous models considered only one location, while the intent of this model was to consider risk of invasion across Florida. Temperature is modeled with an annual cycle of daily mean temperatures. We analyzed historical yearly means and amplitudes and determined that, for this set of weather station data in Florida, means and ranges are correlated (Figure S1, r = −0.96). Therefore, we used the annual mean temperature (*T_mean_*) as a parameter (Table 1) in sensitivity analyses, then calculated *T_range_* using a linear equation fit to the data,

(1) $$T_{\begin{array}{l} range \end{array}}=42.05-1.3*T_{mean}$$

daily temperature was then calculated as

(2) $$T_{day}=\left(\frac{T_{range}}{2}\right)*\left(-cos\left[\frac{2\pi }{365}(t-26.5)\right]\right)+T_{mean}.$$

Day 0 of the simulation is 1 January; the offset in the cosine function (26.5) aligns the temperature function maximum with historical annual peak temperatures. *T_mean_* was varied to reflect spatial and temporal variation in Florida (Table 1).

#### 2.1.2. Human Population

To focus attention on the dynamics of the mosquito–virus interaction, the human population was simplified. Human populations are relatively stable over short time periods, so the human population size was fixed at the initial value (*H_tot_*) and there were no births, immigration, mortality, or emigration. Although neuroinfection and deaths have been reported [38], CHIKV frequently has a low fatality rate [39] so disease-induced mortality was not included. Human population size does vary in communities across Florida, and the size of the human population interacting with a mosquito population is often unknown and dynamic. To focus attention on the differences in the mosquito populations, human population size was fixed at 50,000 in this analysis. The mosquito population size (see below) is described as mosquitoes per human host per year. No latent period in humans was included, as this was thought to be relatively short [39] and, with no human mortality, would act only to slightly slow or delay the dynamics. The duration of infectiousness in humans may extend beyond the symptomatic period (approximately 1 week [40]), and more recently has been suggested to last longer than in other arboviruses [41]). In addition, recent work suggests variability between people and PCR detection of virus for 4–16 days [42]). To focus attention on the mosquito elements of the model, for this analysis duration of infectiousness was set at 8 days.

#### 2.1.3. Mosquito Population Dynamics and Seasonality

Mosquito dynamics are highly variable, with both environmental and biotic influences. To focus on the mosquito–virus interactions, we simplified mosquito dynamics. We assumed that water availability would be the primary driver of recruitment to the adult population, and that both species would respond similarly. This model forces seasonal recruitment using repeated year-round pulses (*iv* days apart) and two large peaks (~N(*q_i_, σ_i_*); *i =* [1,2] for spring, summer peaks) in adult recruitment ([19], Figure S2). Abundance is specified as *ρ_max,j_,* the number of adult female mosquitoes of species *j* per human host over the entire year. The total number of mosquitoes of species *j* is then given by *R_tot,j_* = H_tot_*ρ_max,j_ where *H_tot_* is the number of humans, and is partitioned between the pulses and peaks by the proportion in the baseline pulses (*p_base,j_*) and in the first peak (*p_δ1,j_*); the proportion in the second peak is thus

(3) $$p_{\delta 2,j}=1-p_{base,j}-p_{\delta 1,j}.$$

Recruitment to the adult population was thus (for each mosquito species *j*, *j* = [*aegypti, albopictus*]):

(4) $$\rho_{j}(t)=\rho_{b,j}+p_{\delta 1}R_{tot,j}\delta_{1}+[1-(p_{base}+p_{\delta 2})]R_{tot,j}\delta_{2}$$

where

(5) $$\delta_{i}(t)=\frac{1}{2\pi \sigma_{i}}exp\left[\frac{-{\left(q_{i}-t\right)}^{2}}{2\sigma_{i}{}^{2}}\right]$$

(6) $$\rho_{b,j}(t)=\frac{p_{base}R_{tot,j}}{iv}.$$

#### 2.1.4. Mosquito Mortality

**Figure 2:**
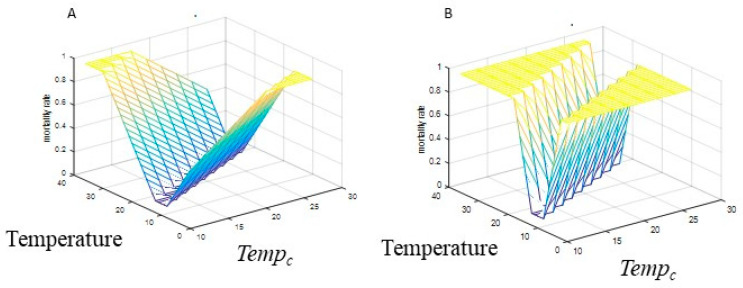
Mortality functions. (**A**) *µ_sl_* = 0.05; (**B**) *µ_sl_* = 0.15.

The previous models used a simple linear increase in mortality with temperature over a relatively narrow temperature range. To include a wider temperature range, increases in mortality at colder temperatures needed to be considered. One study showed relatively little change in mortality over a wide temperature range for *Ae. aegypti* [27], increasing outside the optimal range. Laboratory studies may underestimate mortality, and this study did not extend to temperatures likely to be immediately lethal [27]. The authors fit higher order polynomials to their data, with the best fit with a 4th order polynomial. However, the parameters of this polynomial equation are not easily interpreted biologically. We modeled temperature dependent mortality for both species with a piecewise linear function, resulting in low mortality in an optimal survival temperature range and linear increases on each side. The minimum mortality (*µ_min, j_*) and slope with respect to temperature (*µ_sl,j_*) varied separately for each species (*j =* [1, 2]) but the center of the optimum survival temperature range (*Temp_c_*) did not. The optimum survival range was narrow (*Temp_c_ ± W*; *W* = 2 °C) to allow more variation in the slope while still reaching high levels of mortality (Figure 2). The mortality rate was bounded (*µ_min_*, 1) although the maximum of 1 may not be reached under all parameter combinations. Thus,

(7) $$\mu_{j}(t)=\left(\begin{array}{ccc} \mu_{min,j}-\mu_{sl,j}(Temp-Temp_{c}+2) & & Temp<Temp_{c}-2\\ \mu_{\min,j} & & Temp_{c}-2<Temp<Temp_{c}+2\\ \mu_{min,j}+\mu_{sl,j}(Temp-Temp_{c}-2) & & Temp>Temp_{c}+2 \end{array}\right)$$

#### 2.1.5. Virus Development in Mosquitoes and Vector Competence

The mutations in CHIKV that allow increased vector competence in *Ae. albopictus* affect the EIP (rate of viral development) and the proportion of insects that can acquire and transmit the virus [4]. Virus development in mosquitoes, or the duration of the EIP, is well known to be temperature dependent (e.g., DENV, [16,43,44]; West Nile virus, [45]; CHIKV, [46,47]), and there was evidence that virus development is faster for CHIKV than for some other *Aedes*-transmitted arboviruses [46,48]. We retained the previous structure of a linear increase with temperature and added a lower bound of 10 °C, below which no development occurs. Parameters for this equation vary between mosquito species and are given by *γ_22,j_* and *γ_sl,j_*, virus development at 22 °C, and the slope of the line, respectively. The EIP is then *1/γ_j_* and thus is temperature dependent and can vary between mosquito species. The ability of the mosquitoes to acquire (*b_j_*) and transmit the virus (*β_j_*) is species-specific and varied independently. In this analysis, the vector competence parameters were not correlated, to assess their independent effects on dynamics.

#### 2.1.6. Model Equations

The model structure consists of nine differential equations following changes in susceptible, infected, and recovered humans and susceptible, exposed (latently infected) and infectious mosquitoes of each species (*j*):

As detailed above, mosquito recruitment, mortality and virus development are temperature and thus time dependent but not formally noted in Equation (8) as such for clarity.

In a deterministic model, a virus can persist at extremely low levels where, in a stochastic situation, it would have a high probability of going extinct. To address this, if the combined virus prevalence went below 0.5 (i.e., $H_{in}+\sum_{j}(L_{j}+\;Y_{j})<0.5$) the virus was assumed to die out and all state variables including infection reset to 0.

(8) $$\begin{array}{c} \frac{dH_{s}}{dt}=-H_{s}\left[\sum_{j=1}^{2}\frac{a_{j}b_{j}}{H_{tot}}*Y_{j}\right]\\ \frac{dH_{in}}{dt}=H_{s}\left[\sum_{j=1}^{2}\frac{a_{j}b_{j}}{H_{tot}}*Y_{j}\right]-r_{H}H_{in}\\ \frac{dH_{r}}{dt}=r_{H}H_{in}\\ \frac{dS_{j}}{dt}=\rho_{j}-\mu_{j}S_{j}-a_{j}\beta_{j}(\frac{H_{in}}{H_{tot}})S_{j}\\ \frac{dL_{j}}{dt}=a_{j}\beta_{j}(\frac{H_{in}}{H_{tot}})S_{j}-\gamma_{j}L_{j}-\mu_{j}L_{j}\\ \frac{dY_{j}}{dt}=\gamma_{j}L_{j}-\mu_{j}Y_{j}\\ H_{tot}=H_{s}+H_{in}+H_{r} \end{array}$$

### 2.2. Parameter Values and Sensitivity Analysis

Latin hypercube sampling (LHC), a stratified random sampling method (e.g., [17,18,49]) was used for sensitivity analysis. Briefly, for each parameter of interest a probability density function (pdf) is chosen. The probability axis is divided into *Z* equi-probable intervals and one value chosen from each interval. These probabilities are then back-transformed into parameter values. Values for each parameter are randomly resorted into *Z* parameter sets used to run the model; here *Z* = 250. We used uniform pdfs where little information was available or the intent was to extend over biologically or environmentally feasible ranges, defining the maximum and minimum values. Triangular distributions were used when data were available to identify a most probable value (peak of the distribution) along with maximums and minimums. A preliminary LHC analysis was conducted and showed very high virus activity; parameters were adjusted to reduce outbreak activity to allow analysis and reflect observations of infrequent establishment (few observed local transmission events relative to observed travel-associated imported cases). Distributions and parameter values are given in Table 1 and discussed below, along with fixed values for parameters not varied in this analysis. In addition to this analysis, *Temp_c_* (center of optimal survival range) was varied systematically, (10, 16, 22) repeating the same LHC parameter sets with each *Temp_c_* value (for a total of 750 simulation runs). For each LHC set (each value of *Temp_c_*), epidemic presence or absence was analyzed using multiple logistic regression and the size of the epidemic (*MaxH_i_*) analyzed using multiple regression. Parameters were ranked by the *p*-value of the coefficients and the top five parameters identified as factors of interest and compared between *Temp_c_* values.

LHC and other variations on randomized sampling are unlikely to include sets of parameter values of interest, for example comparing outcomes when a virus strain adapted to one mosquito species is introduced into a mosquito population dominated by that species or another. To consider these events, eight sets of parameter values (see Figure 6 for parameter values) were developed to compare introductions of specific virus strains into defined mosquito populations. For this analysis, human population size was reduced to 5000. Adaptation between viral strain and mosquito species was defined as higher vector competence (*b_j_* and *β_j_*). To limit the parameters considered, EIP was not varied in this comparison. Mosquito populations with varied proportions of the two species and selected mosquito biology parameters, either identical or species-specific (*a_j_,* *µ_min,j_*, *µ_sl,j_*), were modeled and the outcome of introductions assessed. The size of the epidemic was compared between parameter sets. Baseline model runs had mosquito biology parameters set identically between the species. The baseline outcomes were compared to outcomes with species-specific population sizes and vector competence.

#### 2.2.1. Mosquito Population Dynamics and Seasonality

We assume the two species respond similarly to water (rainfall, human generated water) and temperature for hatching and larval development and thus the seasonality of recruitment to the adult population will be similar. However, population abundance of both species varies across space and time in Florida (e.g., [29,31]) and one goal was to consider how this spatio-temporal variation in relative population sizes affects the likelihood of local transmission. Therefore, parameters describing seasonality (*iv,* *q_i,_ σ_i_* (*i =* [1, 2]) were set equal for the two species, while parameters affecting relative abundance were allowed to vary between species. Diapause was not considered, although diapause in *Ae. albopictus* varies with latitude [50]. The two seasonal peaks occurred on days 165 and 245 (*q_1_*, *q_2_*, respectively) with *σ_i_* = [7, 15]. To consider variation in wetting events in the dry season, *iv* was included in the LHS analysis and ranged from 10 to 50 days. Reflecting the rainy season in Florida (approximately June through October) but also considering human generated water in containers (e.g., irrigation, watering plants) and differential response of the two species to desiccation [51], the proportion of mosquito recruitment in the year-round baseline pulses was fixed at *p_base,j_* = [0.13, 0.15] [j = *aegypti, albopictus*] and in the spring peak at *p_δ1,j_* = [0.25, 0.2]. Recruitment to each population, *ρ_max,j_* was sampled independently with a triangular distribution, over a wide range (100–20,000 mosquitoes/species/host/year). This resulted in both the total number of mosquitoes and the relative population size of the two species varying between runs in the sensitivity analysis. Note that although seasonality in recruitment was the same for the two species, temperature-dependent adult mortality was not (see above, [52]). Therefore, adult populations could show differences between species in seasonal abundance. For these and other mosquito biology parameters, the goal was to consider effects of variation expected across different environments and seasons and how differences in the two species may affect transmission.

For the virus strain analysis, recruitment to each population was varied to produce populations that were 0.998:0.002, 0.002:0.998, or 0.5:0.5 *aegypti*:*albopictus*. The parameter values to generate these ratios were: total *ρ_max_ =* 5000/host/year; equal recruitment: *ρ_max_ =* 2500/species; asymmetric recruitment: larger population *ρ_max_ =* 4990, smaller population *ρ_max_ =* 10.

#### 2.2.2. Mosquito Mortality

Although there are numerous studies of mortality rates in both species (reviewed in [52]), the relationship between laboratory measurements and field mortality is poorly understood. We assume field mortality rates will be higher, and therefore extend the range of observed mortality rates to include higher values. Temperature dependence is well documented in the lab, although less so in the field. For both species, the minimum mortality was fixed (*µ_min,j_* = [0.1, 0.06]) with higher mortality rates in *Ae. aegypti*. The slope of mortality with respect to temperature (*µ_sl,,j_*) was included in the sensitivity analysis, with the same range (0.05–0.15) used for both species but values chosen separately, so in each of the LHS runs the species would differ in their sensitivity to temperature. We then systematically varied the central point of the temperature range with minimum mortality (constant between species) over three temperatures (*Temp_c_* = 10, 16, 22), repeating the LHC parameter sets.

For the virus strain analysis, *Temp_c_* = 10 and 22 only. Minimum mortality was set higher in *Ae. aegypti* than in *Ae. albopictus* to reflect shorter expected life span, but temperature sensitivity was lower in *Ae. aegypti* as it is expected to be less sensitive to high temperatures (*µ_min,j_* = [0.08,0.06] and *µ_sl,j_* = [0.006, 0.08]).

#### 2.2.3. Biting Rate

The biting rate, *a_j_*, (bites on humans per day) is the inverse of days between blood meals on humans, *α_j_*. Non-human hosts are not explicitly modeled but included by adjusting *a_j_. Aedes aegypti* can feed more frequently (e.g., [53,54]) and is more likely to feed on humans (e.g., [33,34]), while *Aedes albopictus* can be more opportunistic (e.g., [35]) and so may feed on humans less frequently. However, high variability between populations, particularly for *Ae. albopictus,* has been observed. Thus, *α_j_* is higher and has a wider distribution for *Ae. albopictus* than for *Ae. aegypti*, with triangular pdfs used for both to concentrate sampling near the most probable value (*Ae. aegypti* range 1–5, center 3; *Ae. albopictus* range 2–10, center 5). The model was constructed to facilitate later inclusion of explicit host preference [17,18] or seasonal shifts in host feeding behavior, but was constrained to *a_j_* = 1/*α_j_* for this analysis. LHC sampling was done on *α_j_* then back-transformed for simulation.

In the virus strain analysis, the shorter interval between blood meals for *Ae. aegypti* was retained, with *α* = 3d and for *Ae. albopictus α* = 4d. The difference between the species was decreased from the most probable values (above) to focus attention on the virus transmission parameters (below).

#### 2.2.4. Vector Competence and Virus Development in Mosquitoes

Studies of the vector competence and virus development of these two species and CHIKV strains show variable results and a range of competency (10–80% transmitting virus; e.g., [4,46,55]). Some CHIKV strains have acquired mutations that increase the competence of *Ae. albopictus* (although *Ae. aegypti* may still be the more competent vector). The ability of the mosquitoes to acquire and transmit the virus (*b_j_*) is therefore species-specific. There is less information available on the rate of human infection following infectious bites (*β_j_*), so the range and most likely values for *β_j_* were set the same as *b_j_* but varied independently in the LHC sensitivity analysis. Preliminary analysis with ranges including higher transmission rates reported in the literature generated high levels of outbreaks, restricting analysis and not reflective of the low rate of local transmission following introductions. Accordingly, rates were adjusted down for this analysis (Table 1), while retaining wide ranges to explore the effect on transmission dynamics. Later work supports the wide range of transmission values, with geographic, species, and virus strain variation (e.g., [56]). Notable for CHIKV is the short extrinsic incubation period relative to other arboviruses (e.g., [4,46,48,55]). Virus development in mosquitoes is temperature dependent in other systems (e.g., DENV, [16,43]; West Nile virus, [45]). Limited information available during parameter estimation suggested CHIKV development would also be temperature dependent (e.g., [46]). Therefore, we assumed the CHIKV development rate was a linear function of temperature. Parameters for the linear function are *γ_22_* (virus development at 22 °C, fixed at 0.25 for both species) and *γ_sl_* (slope with respect to temperature, ranging from 0.004 to 0.02 with a most probable value of 0.015 for each species, chosen independently).

In the strain analysis, our goal was to explore the potential effects of virus-mosquito adaptations leading to higher transmission. Therefore, *b*, *β* parameters were varied to reflect different CHIKV strains (high competence [*b*, *β*] = [0.65, 0.9], low competence = [0.5, 0.75]). Virus development parameters *γ_22_* and *γ_sl_* were not varied in this analysis and were fixed at 0.25 and 0.015, respectively, for both species.

The virus was introduced by one infectious human, on the day specified by *t_crjt_*, as this is considered a common route of introduction for CHIKV [57]. The day of introduction spanned most of the year with a uniform distribution, to consider the outcome of introduction at any point in the temperature cycle. This is plausible for introductions, as outbreaks elsewhere and travel occur throughout the year. Day of introduction was not varied in the strain analysis and was fixed at day 200.

The model was programmed in Matlab (The Mathworks, Natick, MA, USA) using the differential equation solver function ode45. Model analysis was conducted in Matlab using multiple regression functions to generate output needed. Matlab code for the ODE model is provided in the Supplemental Materials.

#### 2.2.5. Outcome Variables

The peak number of human cases (*MaxH_i_)* was determined for each simulation run and those runs where *MaxH_i_* > 2 were scored as epidemic (*epi* = 1) or not (*epi* = 0). Further outcome variables were analyzed for epidemic runs only. The time of the peak in humans (*tMaxH_i_*) and the maximum number of humans infected (*MaxH_i_*) were determined for each simulation. The lag between virus introduction and the time of peak virus activity was calculated as *lag =* (*tMaxH_i_* – *t_crit_*).

#### 2.2.6. Analysis

Logistic regression was used to determine the effect of input parameters on the presence/absence of epidemics in the LHC parameter sets, separately for each value of *Temp_c_*. Only main effects were considered, with the full regression model. Parameters were ranked using *p*-values and the five most influential parameters compared between *Temp_c_* values. Given the deterministic model format, all parameters will influence outcomes and so conventional statistical significance is not appropriate; *p*-values are a convenient way to rank the input parameters in importance. Multiple regression was used to assess input parameter effects on peak case numbers (*MaxH_i_*) and *lag* for epidemic runs only, with the most influential parameters identified as for *epi*.

## 3. Results

### 3.1. Epidemic Behavior

**Figure 3:**
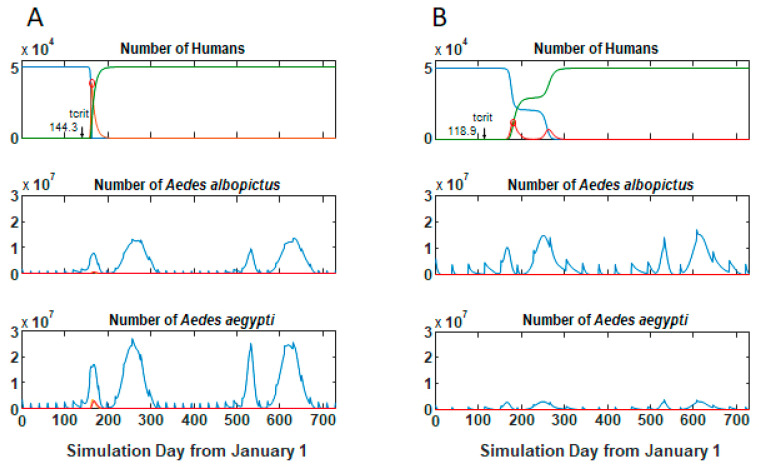
Model behavior. Two simulation runs with different parameters showing (**A**) single and (**B**) bimodal outbreaks. Top panels, number of humans in each class, lower panels, number of adult female mosquitoes of each species in each class. (**A**) Run 67, (**B**) run 249. Parameters given in Table S6. *Temp_c_* = 22 °C. Blue lines, susceptible; red lines, infectious humans or mosquitoes; and green lines, recovered humans. Mosquito panels set to the same Y-axis scaling for comparison; infectious mosquitoes not always visible at this scale.

**Figure 4:**
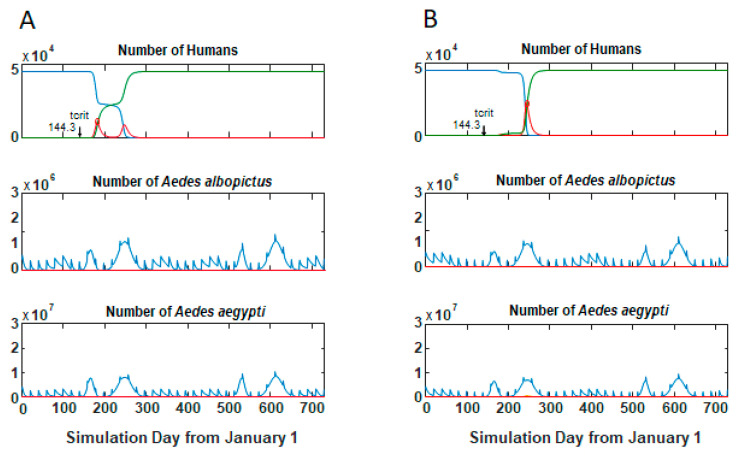
Change in behavior with *Temp_c_*. Other parameter values as for Figure 3A. (**A**) *Temp_c_* = 16 °C. (**B**) *Temp_c_* = 10 °C; compare to Figure 2A. Decreasing *Temp_c_* changes model behavior from rapid single outbreak to bimodal outbreak, then delayed single outbreak. Blue lines, susceptible; red lines, infectious humans or mosquitoes; and green lines, recovered humans.

Most simulation runs demonstrated one of three behaviors: no outbreak and no local transmission occurred, a single outbreak occurred that infected all susceptibles in a relatively short time period (Figure 3A), or two outbreaks occurred (usually relatively close together in time) that also ultimately infected all hosts (Figure 3B). Timing of the outbreaks varied within these behaviors (Table 2). A few parameter sets showed intermediate or alternate behaviors. Changes in *Temp_c_* alone could change this behavior. For example, in one parameter set the outcome was a single epidemic fairly soon after introduction with *Temp_c_* = 22 °C (Figure 3A). Decreasing *Temp_c_* to 16 shifted the outcome behavior to two outbreaks (Figure 4A), while decreasing *Temp_c_* further to 10 recovered a single epidemic peak but with a delay in the time of the epidemic (Figure 4B). The number of parameter sets exhibiting outbreaks increased with *Temp_c_*, from 75 (30%) to 178 (72%) (Table 2).

**Figure 5:**
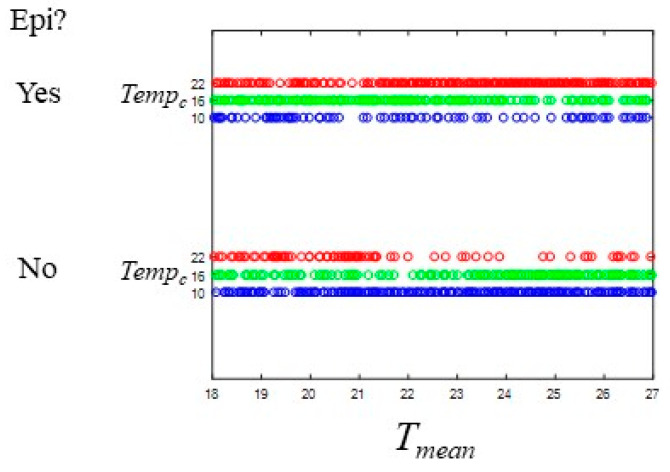
Relationship between epidemic occurrence and mean annual temperature (*T_mean_*). All three values of *Temp_c_* shown (22 °C, red; 16 °C, green; 10 °C, blue). Top set, epidemic occurred; bottom, no epidemic. Each point is one simulation plotted for the *T_mean_* from that run set. Other parameters varied in the LHC sampling scheme. Epidemics were more likely at *Temp_c_* = 22 °C and with higher values of *T_mean_*.

Logistic regression with main effects only had low predictive ability for the likelihood of epidemics (Table 2), suggesting that interactions are important in predicting model outcome. The parameters most influencing the likelihood of epidemics changed based on the value of the central temperature for the mortality function (*Temp_c_*) (Table 3). The annual mean temperature was a key parameter in the likelihood of epidemics at all *Temp_c_* values, but the sign of the coefficient changed (Table 3 and Table S1; Figure 5). When *Temp_c_* was lower (=10 or 16), decreasing annual mean temperature increased the likelihood of epidemics. Conversely, when *Temp_c_* = 22, decreasing annual mean temperature decreased the likelihood of epidemics. Increasing *Ae. albopictus* population size increased the likelihood of epidemics at the lowest *Temp_c_* but decreased it at the highest *Temp_c_*, while population size in *Ae. aegypti* increased the likelihood at middle and high levels. Overall, *Ae. aegypti* parameters were more important than *Ae. albopictus* parameters at all *Temp_c_* levels. Only *Ae. aegypti* transmission rates were in the top five, and only for low and middle *Temp_c_* levels. Similar effects were observed for biting frequency, but with a negative relationship indicating that lower biting frequency of *Ae. aegypti* increased the likelihood of epidemics at lower *Temp_c_* levels. The slope of the mortality rate–temperature relationship for *Ae. aegypti* was in the top five parameters for the middle and high *Temp_c_* levels, with increasing slopes decreasing the likelihood of epidemics.

### 3.2. Size and Lag Time of Epidemics

One measure of epidemic activity is the number of human cases at the peak of the epidemic (*MaxH_i_*). This is influenced by the model behavior and duration of virus activity, as some simulations showed single, short outbreaks (Figure 3) while others had prolonged, lower viral activity. The average *MaxHi* increased with *Temp_c_*, but the range was large in all three sets (Table 2, Figure S3). Adjusted R_2_ values again were low, indicating that interactions are likely to be important (Table 2). Parameters most influencing *MaxH_i_* varied between *Temp_c_* LHS sets (Table 4 and Table S2). At the warmest *Temp_c_*, increased mean annual temperature and later day of introduction both decreased *MaxH_i_*, but this was not observed in the other sets. *Aedes albopictus* traits were important at the coldest *Temp_c_*, but became less important as *Temp_c_* increased. *Aedes aegypti* traits were important at all *Temp_c_* values. Unlike the likelihood of epidemics, mosquito mortality parameters did not strongly affect *MaxH_i_*. Peak activity occurred faster (lower lag between *t_crit_* and *tMaxH_i_*) at the highest *Temp_c_*, but again variation was high (Table 2). The most influential parameters varied between *Temp_c_* LHC sets, but again the main effects regression models had low predictive power (Table 2 and Table 5). The effect of mean annual temperature varied, decreasing lag at low *Temp_c_* but increasing lag at higher *Temp_c_*. At the higher *Temp_c_* values, the population size of both species was important and had a negative effect on *lag*, while at the coldest temperatures, transmission parameters had more effect (Table 5 and Table S3).

### 3.3. Comparisons of Parameter Sets Across Temp_c_

Comparing individual parameter sets across *Temp_c_* values, the general pattern was increasing likelihood of epidemics as *Temp_c_* increases (Table S4). In 60% of parameter sets the outcome was not epidemic at *Temp_c_* = 10, then was epidemic only at *Temp_c_* = 16 (12%), only at *Temp_c_* = 22 (32%), or both (16.4%). We did not observe any parameter sets epidemic at the coldest and warmest temperatures but not epidemic with the middle *Temp_c_* temperature. Decreasing likelihood of epidemics with increasing *Temp_c_* occurred rarely, with 4.4% of parameter sets epidemic only at *Temp_c_* = 10 and 2.8% epidemic at *Temp_c_* = 10 and 16 but not 22.

The peak size of the outbreak also typically increased with *Temp_c_*. Of 57 parameter sets epidemic at all three values, the size increased as *Temp_c_* increased in 84% (Table S5). With parameter sets showing epidemics at only two values of *Temp_c_*, the peak was higher at the warmer *Temp_c_* in 77% of the sets.

### 3.4. Comparison of Strain-Species Combinations

**Figure 6:**
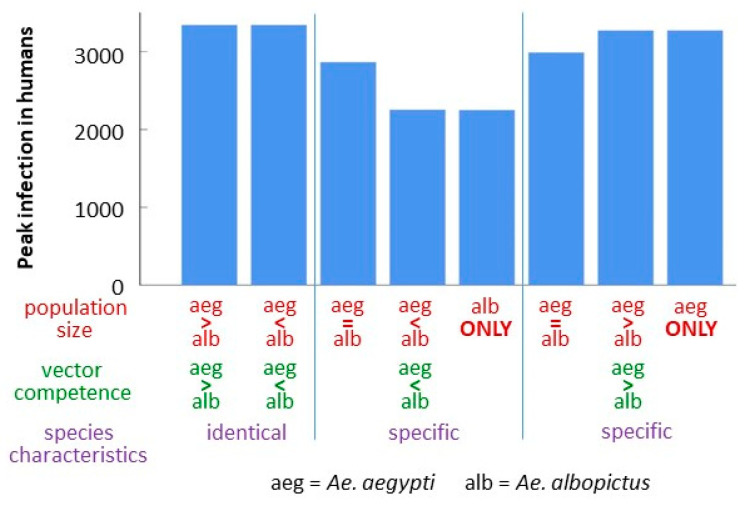
Number of human cases at the peak with species and strain specific parameters. Total mosquito recruitment: *ρ_max_ =* 5000/host/year. Equal recruitment (*aeg = alb*)*: ρ_max_ =* 2500/species. Asymmetric recruitment (*aeg > alb* or *alb > aeg*): high *ρ_max_ =* 4990, low *ρ_max_ =* 10. *b*, *β* parameters varied to reflect different Chikungunya virus strains ([*b*, *β*] = [0.65, 0.9] for species with greater vector competence; [0.5, 0.75] for lower). Species characteristics (“specific”) varied were days between human bloodmeals and mortality rates (*Ae. aegypti: α* = 3d, *µ_22_* = 0.08, *µ_sl_ =* 0.006; *Ae. albopictus: α* = 4d, *µ_22_* = 0.06, *µ_sl_* = 0.08). *Ae. aegypti* parameter values were used for identical species characteristics. All runs: *γ_22_ =* 0.25, *γ_sl_* = 0.015, *T_mean_=* 22 °C, *t_crit_* = 200, *Temp_c_* = 22 °C.

Baseline model runs with species characteristics set identically (functionally one large population) showed identical outbreak sizes regardless of the vector competence assigned to each population and the relative population sizes (Figure 6). When species-specific parameters are used, an *aegypti*-adapted strain shows the same outbreak sizes with only *Ae. aegypti* populations and with *Ae. aegypti*-dominated populations. Equal population sizes of both vectors reduces outbreak size slightly. However, with an *albopictus*-adapted strain (in transmission parameters only), a different pattern is observed. Highest outbreak sizes are lower than those observed with identical species characteristics or with *aegypti*-adapted virus strains, and increasing the proportion of *Ae. albopictus* in the population further decreases outbreak size.

## 4. Discussion

Sensitivity analysis of model outcomes can include systematic variation of individual parameters or randomized methods such as the LHC method. Here, we show that systematic variation of one individual parameter could change the relative ranking of other parameters in an LHC analysis, in addition to changing model behavior. Although it is generally not possible to vary all parameters in a model analysis, this highlights the need to consider the impact of fixed parameters and to identify the values used in any analysis. Even if chosen by fitting to a particular data set, fixed parameters may vary in a broader context so model results should be interpreted with caution outside the conditions set for a specific analysis.

Here, we showed changes in the relative importance of other parameters with variation in one parameter, the center of the temperature range for minimal mosquito mortality. Notably, when this parameter was higher, the slope of the temperature–mortality relationship for *Ae. aegypti* was identified as an important factor in likelihood of epidemics. Interestingly, the same parameter for *Ae. albopictus* did not have a similar effect, and it did not have strong effects on the size of an outbreak for either species. We used a broad range for *Temp_c_*, likely extending beyond biological realism for *Ae. aegypti. Aedes aegypti* is a more tropical mosquito and mortality is likely to be high at lower temperatures. Extending the upper range of this parameter would be of interest, given the increase in the likelihood of invasion at the higher value and the relationship with the mean annual temperature. Future work will consider more specific mortality relationships and broader ranges.

Adult mosquito mortality has been identified as an important parameter in other arbovirus models, even with constant mortality rates or simple relationships with temperature. Complex relationships between mortality and temperature have been observed for *Ae. aegypti*, but less is known about *Ae. albopictus*. Clearly a better understanding of the factors influencing mortality, and improving inclusion of these complexities in arbovirus transmission models, is needed.

Our results support the general consensus that *Ae. aegypti* is more important as a vector in CHIKV dynamics in many environments, but *Ae. albopictus* can support transmission and outbreaks either alone or in mixed populations with low *Ae. aegypti* abundance. Interestingly, even with a viral strain adapted to *Ae. albopictus, Ae. aegypti* supported higher transmission (Figure 6). However, this analysis did not include changes to the EIP known to occur with some mutations, and this should be considered further. In our analysis, transmission rates (*b* and *β*) were consistently identified as key factors, while virus development rates (*γ*) had limited impacts on outcome variables, suggesting that mutations affecting transmission rates may be more important. The virus may acquire adaptive mutations during an outbreak, and including variation in key parameters over time may be important in understanding how this would affect virus dynamics. In this model, the higher transmission with *Ae. aegypti* likely depended on the species-specific host preferences (higher biting rates on humans). The more opportunistic feeding patterns of *Ae. albopictus* have been reported from multiple locations, but there may also be situations where human blood meals are more frequent. In the field, host preference may be dynamic and realized feeding dependent on host availability, host preference [37,58,59,60], and the degree to which both species exhibit gonotrophic discordance (e.g., [53,61]). Additional exploration of the model including variation in human biting rates over time would be of interest to better understand the potential role of *Ae. albopictus* in CHIKV local transmission.

Other models of CHIKV have shown similar results in some aspects, while diverging in others. Manore et al. [20] developed a model to compare CHIKV and DENV transmission with either *Ae. aegypti* or *Ae. albopictus* vectors, but did not include mixed mosquito populations, seasonal mosquito dynamics, or temperature dynamics. Some of the same parameters were identified as having the most influence on the outcome, including mosquito mortality, biting rate, and transmission rates. Overall their model showed much slower dynamics for CHIKV epidemic development after introduction than did ours. This is likely due to the temperature dependence in our model, leading to seasonally changing mosquito mortality rates and duration of the latency stage. Our central transmission rates were lower than the baseline transmission rates of [20] as well, likely slowing the dynamics. Adjusting parameters in our model to approximate those in the Manore et al. model led to similarly slow dynamics (not shown).

Our results support temperature effects on the likelihood, size, and structure of epidemics. However, successful invasions occurred over the full range of mean annual temperatures for Florida, and at all three values of *Temp_c_* (Figure 4). While the interaction between the annual mean temperature and the central temperature for minimum mosquito mortality was complex, the range of conditions with successful invasions implies that all of Florida is at risk of invasion by CHIKV. Other areas that have similar climates and extend over temperate–subtropical zones, such as Brazil, may have similar effects of mean annual temperatures and temperature-dependent parameters. Outbreaks in other temperate areas (e.g., Italy) further illustrate the complex interactions between temperature and transmission dynamics. Extensions of the sensitivity analysis to higher and lower *T_mean_* values would be of interest to explore the risk in other areas. This would, however, require assessment of the relationship between mean and amplitude of the temperature curve, as the relationship used here will not always be appropriate (Salami et al. unpublished). Several input parameters influenced the likelihood of epidemics, but over the ranges considered none showed threshold effects, suggesting that there is a wide range of parameter space that allows outbreaks.

The parameters identified as important in this analysis are also likely to influence other *Aedes*-transmitted viruses such as ZIKV and DENV, suggesting areas for research to predict the outcomes of introductions of these viruses. However, virus-specific parameter estimates, along with location-specific temperature curves, are needed to assess the likelihood of invasion and identify factors that most influence epidemic dynamics.

Adult recruitment patterns were based on rainfall and modeled similarly for both vector species. However, in reality egg diapause in *Ae. albopictus* reduces hatching probabilities of this species in the late fall and early winter periods in Florida [50]. By contrast, egg diapause does not occur in *Ae. aegypti*, which does not experience seasonal hatching interruptions. These recruitment differences could also contribute to the relative involvement of the two species in transmission dynamics. Different seasonal patterns of recruitment affected simulated dynamics of WNV [19] and should be considered in more detail for CHIKV.

With repeated introductions of arboviruses into Florida and elsewhere, we need to improve our understanding of what influences whether an introduction leads to establishment and outbreaks. Mosquito mortality has consistently been identified as important, is expected to vary considerably in the field, and is a target for novel control efforts to mitigate pathogen transmission (e.g., life-shortening microbes, *Wolbachia*, [62], *Beauveria bassiana*, [63]; gene drive systems, [64]). Further exploration of this is needed, including temperature dependency, species and individual variation, and other environmental influences. Stochastic factors in mortality and human–mosquito contact will also play a role in virus establishment, and individual based models allowing this structure would be valuable. Expanded consideration of virus lineages, genotypes, and mutations and interactions with mosquito species will be valuable to better understand the potential for virus invasion and outbreaks. Interactions between parameters are clearly important in model analysis, but it is unclear how this relates to field conditions. Additionally, spatial variation in human-related factors may influence model parameters (e.g., socioeconomic factors [65]). Incorporating interactions and increased variation into modeling studies will improve our ability to understand arbovirus dynamics and target empirical studies on critical factors.

## Figures and Tables

**Table 1:** State variables, parameters, and parameter distributions used in the model and LHC analysis.

Descriptions	Symbol	Distribution ^a^	Range	Center
**State variables**				
Susceptible humans	*H_s_*			
Infectious humans	*H_in_*			
Recovered humans	*H_r_*			
Susceptible mosquitoes, species j	*S_j_*			
Latent mosquitoes, species j	*L_j_*			
Infectious mosquitoes, species j	*Y_j_*			
**Parameters**				
Mean of temperature curve	*T_mean_*	Uni	18–27	
Day virus is introduced	*t_crit_*	Uni	5–360	
Human recovery rate	*r_H_*	Fixed		0.125
Total (initial) number of humans	*H_tot_*	Fixed		50,000
**Both mosquito species**				
Interval between pulses (both species)	*iv*	Uni	10–50	
Mean day peak one (from January 1)	*q_1_*	Fixed		165
Spread peak one	*σ_1_*	Fixed		7
Mean day peak two (from January 1)	*q_2_*	Fixed		245
Spread peak two	*σ_2_*	Fixed		15
Width of optimal survival range	*W*	Fixed		2
Central point of optimal survival temperature range	*Temp_c_*	Set values	[10,16,22]	
***Aedes albopictus***				
Transmission mosquito to human host	*b_alb_*	Tri	0.1–0.7	0.4
Transmission human host to mosquito	*β_alb_*	Tri	0.1–0.7	0.4
Days between blood feeding on humans	*α_alb_*	Tri	2–20	5
Proportion of population in peak one	*p_δ1,alb_*	Fixed		0.2
Proportion of population in pulses all year	*p_base,alb_*	Fixed		0.15
Temperature—mortality slope	*µ_sl,alb_*	Uni	0.05–0.15	
Virus development at 22.5 °C	*γ_22,alb_*	Fixed		0.25
Temperature—virus development slope	*γ_sl,alb_*	Tri	0.004–0.02	0.015
Maximum recruitment	*ρ_max,alb_*	Tri	100–20,000	5000
Total recruitment through year	*R_tot,alb_*	Calculated		
***Aedes aegypti***				
Transmission mosquito to human host	*b_aeg_*	Tri	0.1–0.7	0.4
Transmission human host to mosquito	*β_aeg_*	Tri	0.1–0.7	0.4
Days between blood feeding on humans	*α_aeg_*	Tri	1–5	3
Proportion of population in peak one	*p_δ1,aeg_*	Fixed		0.25
Proportion of population in pulses all year	*p_base,aeg_*	Fixed		0.13
Minimum mortality (at Temp_c_ ± W)	*µ_min,aeg_*	Fixed		0.1
Slope of temperature—mortality line	*µ_sl,aeg_*	Uni	0.05–0.15	
Virus development at 22.5 °C	*γ_22,aeg_*	Fixed		0.25
Temperature—virus development slope	*γ_sl,aeg_*	Tri	0.004–0.02	0.015
Maximum recruitment	*ρ_max,aeg_*	Tri	100–20,000	5000
Total recruitment through year	*R_tot,aeg_*	Calculated		

LHC—Latin hypercube sampling; ^a^ Distributions: Tri, triangular, Uni, uniform.

**Table 2:** Outcome variables for sensitivity analysis at three values of *Temp_c_*.

	Central Temperature, *Temp_c_* (°C)		
Outcome Variable	10	16	22
# Epidemics (out of 250 parameter sets)	75	135	178
Average *MaxH_i_* (for epidemic runs)	13,579	17,174	26,443
*MaxH_i_* range	2.02–34,916	2.02–38,804	3.71–41,621
Average (range) *lag* (time from introduction to *MaxH_i_*, epidemic runs)	63.9 (9.7–286.8)	68 (9.1–373)	37.7 (8.85–139.2)
Logistic regression on epidemics, adjusted R^2^	0.1	0.14	0.12
Regression model for *MaxH_i_* (epidemic runs only), adjusted R^2^	0.5	0.34	0.1
Regression model for *lag* (epidemic runs only), adjusted R^2^	0.2	0.3	0.2

Ranges, means, and regression R^2^ for outcome variables. Details of regression analyses in Table 3, Table 4 and Table 5.

**Table 3:** Top five parameters influencing likelihood of epidemics with different values of the central temperature in the mortality function, *Temp_c_*. Parameters identified using full, main effects logistic regression model and ranked by *p*-value. Negative regression coefficients indicated by (-).

Central Temperature, *Temp_c_* (°C)		
10	16	22
Annual mean temperature (*T_mean_*) (−)	Annual mean temperature (*T_mean_*) (−)	Annual mean temperature (*T_mean_*)
		Day of introduction (*t_crit_*)
*Ae. albopictus* recruitment per host (*ρ_max,alb_*)		*Ae. albopictus* recruitment per host (*ρ_max,alb_*) (−)
	*Ae. aegypti* recruitment per host (*ρ_max,aeg_*)	*Ae. aegypti* recruitment per host (*ρ_max,aeg_*)
Transmission: humans to *Ae. aegypti* (*β_aeg_*)	Transmission: humans to *Ae. aegypti* (*β_aeg_*)	
Transmission: *Ae. aegypti* to humans (*b_aeg_*)		
*Ae. aegypti* biting frequency (*α_aeg_*) (−)	*Ae. aegypti* biting frequency (*α_aeg_*) (−)	
	*Ae. aegypti* mortality–temperature slope (*µ_sl,aeg_*) (−)	*Ae. aegypti* mortality–temperature slope (*µ_sl,aeg_*) (−)

**Table 4:** Top five parameters influencing *MaxH_i_*. Includes only epidemic simulations, main effects only.

Central Temperature, *Temp_c_* (°C)		
10	16	22
		Annual mean temperature (*T_mean_*) (-)
		Day of introduction (*t_crit_*) (-)
Transmission: humans to *Ae. albopictus* (*β_alb_*)		
*Ae. albopictus* biting frequency (*α_alb_*) (-)	*Ae. albopictus* biting frequency (*α_alb_*) (-)	
*Ae. aegypti* recruitment per host (*ρ_max,aeg_*)	*Ae. aegypti* recruitment per host (*ρ_max,aeg_*)	
Transmission: humans to *Ae. aegypti* (*β_aeg_*)	Transmission: humans to *Ae. aegypti* (*β_aeg_*)	Transmission: humans to *Ae. aegypti* (*β_aeg_*)
	Transmission: *Ae. aegypti* to humans (*b_aeg_*)	Transmission: *Ae. aegypti* to humans (*b_aeg_*)
*Ae. aegypti* biting frequency (*α_aeg_*) (-)	*Ae. aegypti* biting frequency (*α_aeg_*) (-)	*Ae. aegypti* biting frequency (*α_aeg_*) (-)

**Table 5:** Top five parameters influencing *lag*. Includes only epidemic simulations, main effects only.

Central Temperature, *Temp_c_* (°C)		
10	16	22
Annual mean temperature (*T_mean_*) (-)		Annual mean temperature (*T_mean_*)
Transmission: *Ae. albopictus* to humans (*b_alb_*) (-)		
Transmission: humans to *Ae. albopictus* (*β_alb_*)		
	*Ae. albopictus* biting frequency (*α_alb_*)	
	*Ae. albopictus* mortality—temperature slope (*µ_sl,alb_*) (-)	
*Ae. albopictus* temperature—virus development slope (*γ_sl,alb_*)		
	*Ae. albopictus* recruitment per host (*ρ_max,alb_*)(-)	*Ae. albopictus* recruitment per host (*ρ_max,alb_*)(-)
	*Ae. aegypti* recruitment per host (*ρ_max,alb_*)(-)	*Ae. aegypti* recruitment per host (*ρ_max,aeg_*)(-)
Transmission: humans to *Ae. aegypti* (*β_aeg_*)		Transmission: humans to *Ae. aegypti* (*β_aeg_*)(-)
	*Ae. aegypti* biting frequency (*α_aeg_*)	*Ae. aegypti* biting frequency (*α_aeg_*)

## Supplementary Materials

The following are available online at https://www.mdpi.com/1999-4915/12/8/830/s1, Figure S1: Relationship between mean and range of yearly temperatures for Florida weather stations. Figure S2: Phases of modeled mosquito populations. Figure S3: *MaxH_i_* histograms for each *Temp_c_*. Figure S4: Relationship between epidemic occurrence and selected parameters. Table S1, Results of logistic regression on epidemic behavior; Table S2, Results of regression on *MaxH_i_*; Table S3, Results of regression on *lag;* Table S4, Increasing epidemics with *Temp_c_*; Table S5, Increasing size of epidemics with *Temp_c_*; Table S6, Parameter values for simulations shown in Figure 3 and Figure 4. Text S1: Model Matlab program.
