# Supplementary material for: Effects of Mosquito Biology on Modeled Chikungunya Virus Invasion Potential in Florida
Source: Viruses. 2020 Jul 30;12(8):830. doi: 10.3390/v12080830 (PMC7472381; doi:10.3390/v12080830)
Supplement: Supplementary file 1 [file viruses-12-00830-s001.zip › viruses-869203-supp.pdf]

Supplementary Materials: Effects of Mosquito Biology on Modeled Chikungunya Virus Invasion Potential in Florida

Cynthia C. Lord, L. Philip Lounibos, Joseph J. Pohedra and Barry W. Alto

Table S1. Results of logistic regression on epidemic behavior.

Temp_c	**10**		**16**		**22**	
**Parameter**	**coefficient**	***p***	**coefficient**	***p***	**coefficient**	***p***
intercept	-3.03	2.32×10^{-1}	5.84	1.25×10^{-2}	-3.61	1.54×10^{-1}
T_{mean}	**-9.53×10^{-2}**	**1.08×10^{-1}**	**-2.54×10^{-1}**	**2.07×10^{-5}**	**2.81×10^{-1}**	**1.93×10^{-5}**
t_{crit}	2.24×10^{-3}	1.43×10^{-1}	1.42×10^{-3}	3.25×10^{-1}	**3.02×10^{-3}**	**5.94×10^{-2}**
b_{alb}	1.40	2.73×10^{-1}	-9.20×10^{-2}	9.38×10^{-1}	-8.56×10^{-1}	5.09×10^{-1}
β_{alb}	-1.93×10^{-1}	8.80×10^{-1}	1.68	1.62×10^{-1}	1.99	1.41×10^{-1}
α_{alb}	-1.33×10^{-2}	7.38×10^{-1}	-4.56×10^{-2}	2.14×10^{-1}	-1.24×10^{-2}	7.53×10^{-1}
$\mu_{sl,alb}$	-2.25	6.72×10^{-1}	-4.27	3.97×10^{-1}	1.95	7.26×10^{-1}
$\gamma_{sl,alb}$	3.12×10^{1}	5.21×10^{-1}	7.23	8.73×10^{-1}	2.07	9.66×10^{-1}
$\rho_{max,alb}$	**1.00×10^{-4}**	**8.35×10^{-3}**	4.70×10^{-5}	1.77×10^{-1}	**-6.70×10^{-5}**	**8.22×10^{-2}**
b_{aeg}	**2.69**	**3.61×10^{-2}**	1.89	1.09×10^{-1}	5.99×10^{-1}	6.42×10^{-1}
β_{aeg}	**5.35**	**6.44×10^{-5}**	**2.58**	**3.13×10^{-2}**	7.35×10^{-1}	5.75×10^{-1}
α_{aeg}	**-3.35×10^{-1}**	**7.76×10^{-2}**	**-4.69×10^{-1}**	**1.05×10^{-2}**	-3.17×10^{-1}	1.09×10^{-1}
$\mu_{sl,aeg}$	-3.76	4.84×10^{-1}	**-1.15×10^{1}**	**2.40×10^{-2}**	**-1.15×10^{1}**	**4.44×10^{-2}**
$\gamma_{sl,aeg}$	1.66×10^{1}	7.20×10^{-1}	-3.14	9.42×10^{-1}	-4.55×10^{1}	3.53×10^{-1}
$\rho_{max,aeg}$	4.50×10^{-5}	2.01×10^{-1}	**1.27×10^{-4}**	**5.35×10^{-4}**	**7.90×10^{-5}**	**3.76×10^{-2}**
iv	-1.33×10^{-3}	9.19×10^{-1}	-2.36×10^{-2}	6.38×10^{-2}	-2.14×10^{-2}	1.21×10^{-1}

Entries in bold are the 5 smallest *p*–values for each $Temp_c$.

Table S2. Results of regression on $MaxH_i$ (epidemic runs only).

Temp_c	**10**		**16**		**22**	
**Parameter**	**coefficient**	***p***	**coefficient**	***p***	**coefficient**	***p***
intercept	-5.49×10^{3}	7.64×10^{-1}	3.60×10^{4}	8.24×10^{-3}	4.40×10^{4}	4.66×10^{-5}
T_{mean}	-4.37×10^{2}	3.11×10^{-1}	-8.61×10^{2}	1.37×10^{-2}	**-9.19×10^{2}**	**1.40×10^{-3}**
t_{crit}	-1.27×10^{1}	3.46×10^{-1}	5.59	5.05×10^{-1}	**-1.99×10^{1}**	**9.88×10^{-3}**
b_{alb}	-4.38×10^{3}	6.24×10^{-1}	7.01×10^{3}	3.01×10^{-1}	4.66×10^{3}	4.10×10^{-1}
β_{alb}	**2.13×10^{4}**	**2.92×10^{-2}**	9.90×10^{3}	1.58×10^{-1}	6.98×10^{3}	2.23×10^{-1}
α_{alb}	**-1.17×10^{3}**	**9.00×10^{-5}**	**-6.61×10^{2}**	**3.77×10^{-3}**	-1.17×10^{2}	4.90×10^{-1}
$\mu_{sl,alb}$	4.46×10^{4}	2.18×10^{-1}	-3.50×10^{4}	2.27×10^{-1}	1.01×10^{4}	6.60×10^{-1}
$\gamma_{sl,alb}$	5.86×10^{5}	1.45×10^{-1}	-1.68×10^{5}	5.16×10^{-1}	-1.13×10^{5}	6.05×10^{-1}
$\rho_{max,alb}$	5.57×10^{-2}	8.36×10^{-1}	2.69×10^{-2}	8.94×10^{-1}	1.68×10^{-1}	3.26×10^{-1}
b_{aeg}	1.46×10^{4}	1.49×10^{-1}	**1.69×10^{4}**	**9.96×10^{-3}**	**1.39×10^{4}**	**1.37×10^{-2}**
β_{aeg}	**3.11×10^{4}**	**1.59×10^{-3}**	**2.07×10^{4}**	**2.46×10^{-3}**	**1.70×10^{4}**	**2.00×10^{-3}**
α_{aeg}	**-7.25×10^{3}**	**2.60×10^{-6}**	**-5.66×10^{3}**	**1.00×10^{-7}**	**-2.31×10^{3}**	**5.44×10^{-3}**
$\mu_{sl,aeg}$	3.96×10^{4}	2.96×10^{-1}	-5.64×10^{4}	5.08×10^{-2}	-4.46×10^{4}	6.11×10^{-2}
$\gamma_{sl,aeg}$	3.50×10^{5}	3.12×10^{-1}	1.06×10^{5}	6.74×10^{-1}	-7.44×10^{4}	7.02×10^{-1}
$\rho_{max,aeg}$	**1.28**	**2.94×10^{-5}**	**9.37×10^{-1}**	**1.20×10^{-6}**	3.72×10^{-1}	2.07×10^{-2}
iv	1.04×10^{2}	3.01×10^{-1}	-6.94	9.23×10^{-1}	-2.19×10^{1}	7.04×10^{-1}

Entries in bold are the 5 smallest *p*–values for each $Temp_c$.

Table S3. Results of regression on *lag* (epidemic runs only).

Temp_c	**10**		**16**		**22**	
**Parameter**	**coefficient**	***p***	**coefficient**	***p***	**coefficient**	***p***
intercept	1.08×10^{2}	3.30×10^{-1}	1.51×10^{2}	6.56×10^{-2}	-1.40×10^{1}	5.61×10^{-1}
T_{mean}	**-5.94**	**2.45×10^{-2}**	-2.26	2.81×10^{-1}	**2.99**	**7.88×10^{-6}**
t_{crit}	-8.69×10^{-2}	2.83×10^{-1}	-7.66×10^{-2}	1.33×10^{-1}	-3.36×10^{-2}	5.64×10^{-2}
b_{alb}	**-1.14×10^{2}**	**3.75×10^{-2}**	-2.17×10^{1}	5.96×10^{-1}	-1.06×10^{1}	4.14×10^{-1}
β_{alb}	**8.56×10^{1}**	**1.40×10^{-1}**	-1.38×10^{1}	7.45×10^{-1}	9.08	4.88×10^{-1}
α_{alb}	-1.62	3.38×10^{-1}	**3.11**	**2.34×10^{-2}**	3.31×10^{-1}	3.95×10^{-1}
$\mu_{sl,alb}$	-1.83×10^{1}	9.33×10^{-1}	**-4.54×10^{2}**	**1.06×10^{-2}**	2.35×10^{1}	6.56×10^{-1}
$\gamma_{sl,alb}$	**3.56×10^{3}**	**1.41×10^{-1}**	1.05×10^{3}	5.01×10^{-1}	-4.27×10^{2}	3.94×10^{-1}
$\rho_{max,alb}$	-5.83×10^{-5}	9.71×10^{-1}	**-4.44×10^{-3}**	**3.93×10^{-4}**	**-9.95×10^{-4}**	**1.18×10^{-2}**
b_{aeg}	3.21	9.57×10^{-1}	-1.02×10^{1}	7.94×10^{-1}	-4.15	7.46×10^{-1}
β_{aeg}	**1.20×10^{2}**	**3.83×10^{-2}**	-2.64×10^{1}	5.16×10^{-1}	**-3.77×10^{1}**	**2.70×10^{-3}**
α_{aeg}	8.27	3.28×10^{-1}	**1.87×10^{1}**	**2.59×10^{-3}**	**5.34**	**4.99×10^{-3}**
$\mu_{sl,aeg}$	-3.13×10^{2}	1.71×10^{-1}	1.41×10^{2}	4.17×10^{-1}	1.12×10^{2}	4.10×10^{-2}
$\gamma_{sl,aeg}$	2.77×10^{3}	1.84×10^{-1}	5.23×10^{2}	7.31×10^{-1}	3.72×10^{1}	9.34×10^{-1}
$\rho_{max,aeg}$	-1.14×10^{-3}	5.06×10^{-1}	**-2.65×10^{-3}**	**1.86×10^{-2}**	**-1.18×10^{-3}**	**1.46×10^{-3}**
iv	3.98×10^{-1}	5.09×10^{-1}	7.73×10^{-2}	8.60×10^{-1}	-6.25×10^{-2}	6.37×10^{-1}

Entries in bold are the 5 smallest *p*–values for each $Temp_c$.

Table S4. Increasing epidemics with $Temp_c$. All possible combinations of not epidemic (0) and epidemic (1) across the 3 sets shown, with the number and proportion of the total runs (250) showing that combination. Within individual run sets, epidemics were more likely as $Temp_c$ increased.

**Epidemic in *Temp_c***				
**10**	**16**	**22**	**#Runs**	**Proportion**
0	0	0	24	0.096
0	0	1	80	0.32
0	1	1	41	0.164
0	1	0	30	0.12
1	0	0	11	0.044
1	0	1	0	0
1	1	0	7	0.028
1	1	1	57	0.228

Table S5. Increasing size of epidemics with $Temp_c$. Possible combinations of $MaxH_i$ across the 3 $Temp_c$ sets for different combinations of *epi* = 1 (epidemic) or 0 (not epidemic), where at least 2 were epidemic. For each combination of presence/absence of epidemics, the size of the epidemic increased with $Temp_c$.

**$MaxH_i$ Relative Size for $Temp_c$ Values**	**#Runs**
**all epidemic (*epi* =1)**	
10 > 16 > 22	2
10 > 16 < 22	2
10 < 16 > 22	5
10 < 16 < 22	48
**10, 16 *epi* =1; 22 *epi* =0**	
10 > 22	2
10 < 22	5
**10 *epi* =0; 16, 22 *epi* =1**	
16 > 22	9
16 < 22	32

Table S6. Parameter values for simulations shown in Figures 3 and 4.

	**run#**	
**Parameter**	**67**	**249**
T_{mean}	19.850	22.746
t_{crit}	144.273	118.893
iv	19.703	37.978
b_{alb}	0.305	0.280
β_{alb}	0.389	0.461
α_{alb}	7.833	14.942
$\mu_{sl,alb}$	0.095	0.083
$\gamma_{sl,alb}$	0.015	0.018
$\rho_{max,alb}$	3786.963	6551.880
b_{aeg}	0.351	0.630
β_{aeg}	0.407	0.309
α_{aeg}	2.090	2.755
$\mu_{sl,aeg}$	0.095	0.114
$\gamma_{sl,aeg}$	0.012	0.008
$\rho_{max,aeg}$	9303.730	1957.701

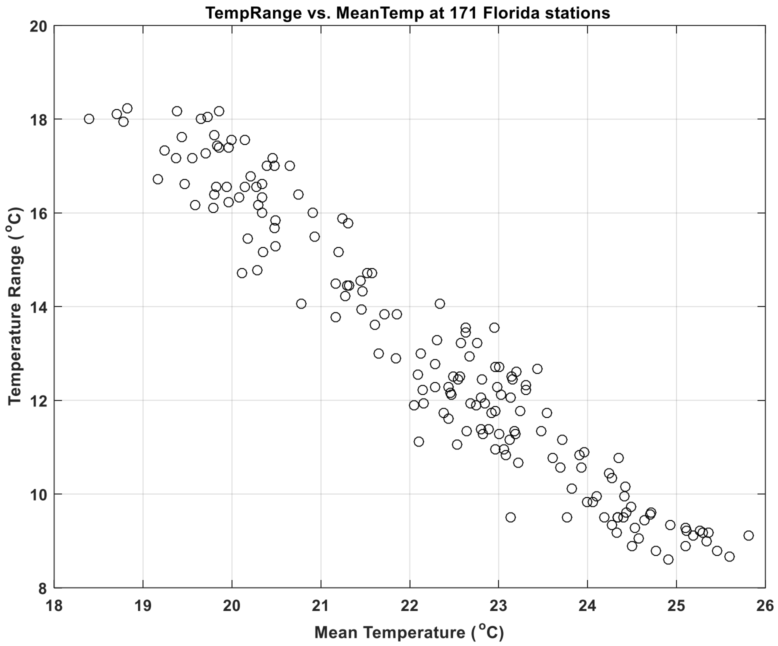

Figure S1. Relationship between mean and range of yearly temperatures for Florida weather stations.

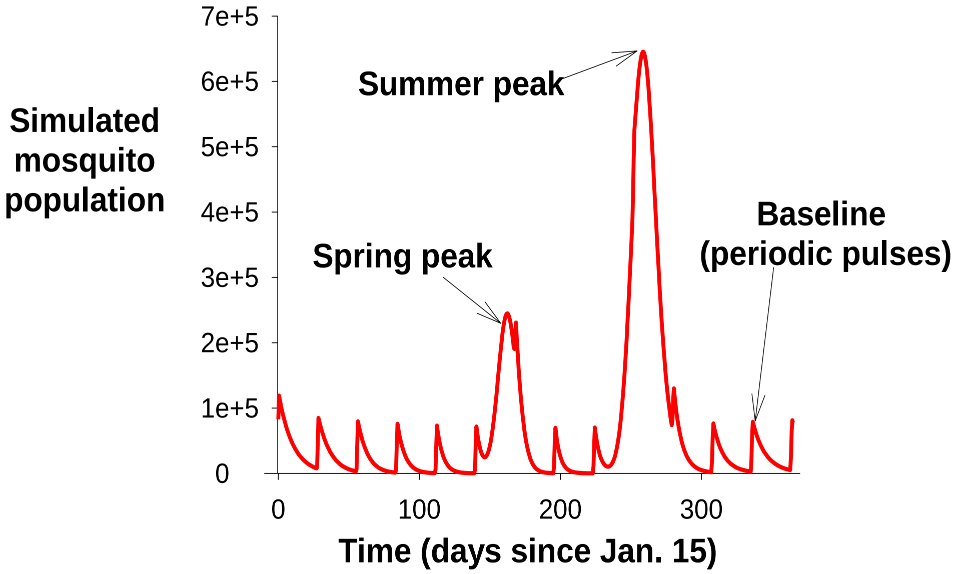

Figure S2. Phases of modeled mosquito populations.

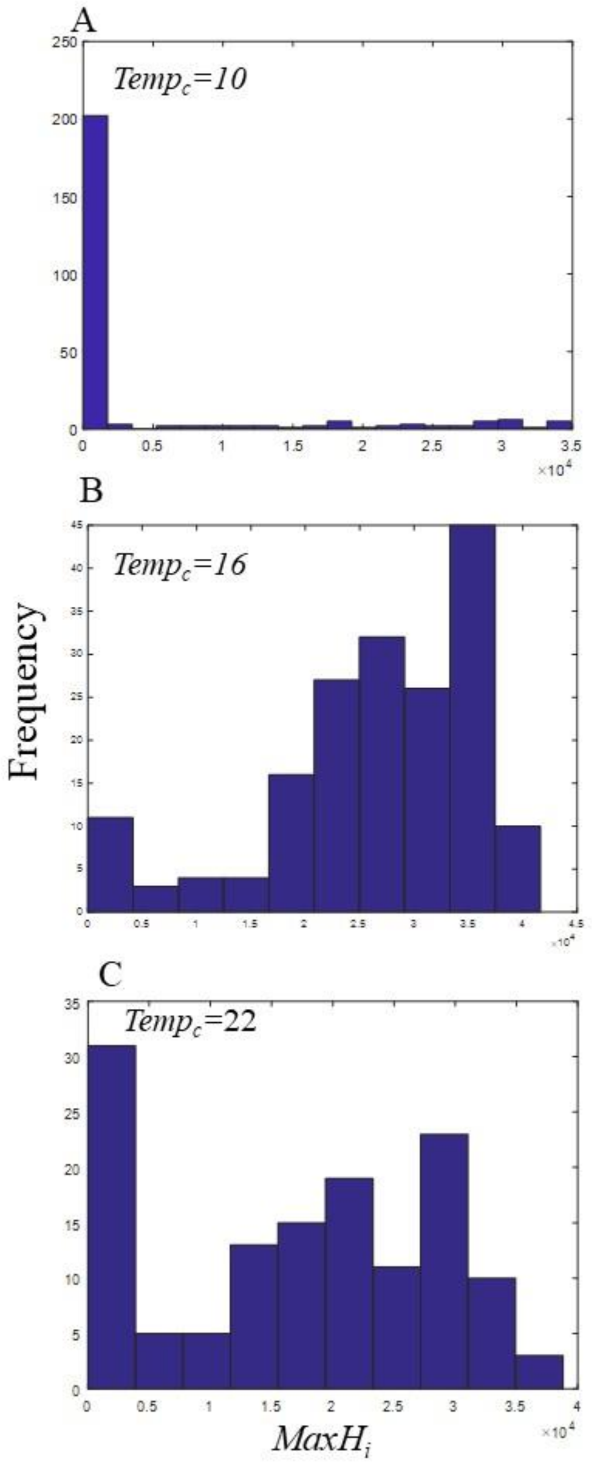

Figure S3. $MaxH_i$ histograms for each $Temp_c$. Note change in y−axis.

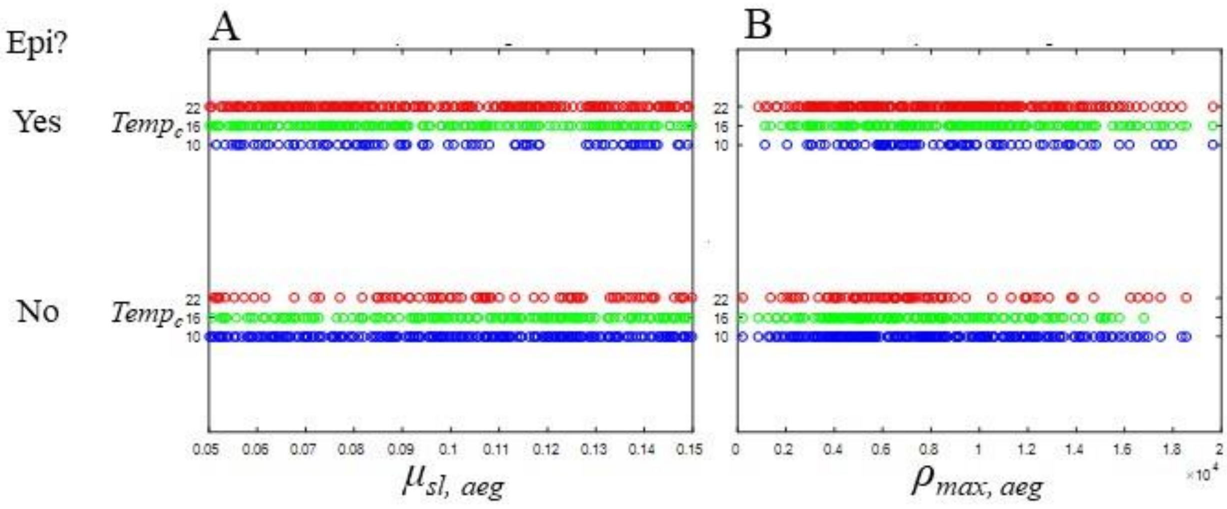

Figure S4. Relationship between epidemic occurrence and A) mortality – temperature slope, B) maximum mosquito population, for *Ae. aegypti.* All 3 values of $Temp_c$ shown (22 °C, red; 16 °C, green; 10°C, blue). Top set, epidemic occurred; bottom, no epidemic. Each point is one simulation plotted for parameter from that run set. All other parameters varied as well, in the LHC sampling scheme. Epidemics were more likely at $Temp_c$ = 22 °C, with lower values of $\mu_{sl,aeg}$ and higher values of $\rho_{max,aeg}$ at $Temp_c$ = 16°C and 22°C.
