# Supplementary material for: Effects of Mosquito Biology on Modeled Chikungunya Virus Invasion Potential in Florida
Source: Viruses. 2020 Jul 30;12(8):830. doi: 10.3390/v12080830 (PMC7472381; doi:10.3390/v12080830)
Supplement: Supplementary file 1 [file viruses-12-00830-s001.zip › Lord_Suppl_Modelcode_rev.pdf]

Parameter mapping between equations and Matlab code

**Descriptions**	**Symbol**	**Matlab**	**Distribution**[a]	**Range**	**Center**
**State variables**					
Susceptible Humans	H_s				
Infectious Humans	H_{in}				
Recovered Humans	H_r				
Susceptible mosquitoes, species *j*	S_j				
Latent mosquitoes, species *j*	L_j				
Infectious mosquitoes, species *j*	I_j				
**Parameters**					
Mean of temperature curve	T_{mean}	Tmean	Uni	18-27	
Day virus is introduced	t_{crit}	Tcrit	Uni	5-360	
Human recovery rate	r_H	recH	Fixed		0.125
Total (initial) number of humans	H_{tot}	initH	Fixed		50000
**Both mosquito species**					
Interval between pulses (both species)	iv	intervalAlb, aeg	Uni	10-50	
Mean day peak one (from January 1)	q_1	mean1Alb,aeg	Fixed		165
Spread peak one	σ_1	sigma1Alb,aeg	Fixed		7
Mean day peak two (from January 1)	q_2	mean2Alb,aeg	Fixed		245
Spread peak two	σ_2	sigma2Alb,aeg	Fixed		15
Width of optimal survival range	W	Hard coded	Fixed		2
Central point of optimal survival temperature range	$Temp_c$	Threshold	Set values	[10,16,22]	
Aedes albopictus					
Transmission mosquito to human host	b_{alb}	bHAlb	Tri	0.1-0.7	0.4
Transmission human host to mosquito	β_{alb}	betaHAlb	Tri	0.1-0.7	0.4
Days between blood feeding on humans	α_{alb}	alphaprimeAlb	Tri	2-20	5

Descriptions	Symbol	Matlab	Distribution[a]	Range	Center
Proportion of population in peak one	$p_{\delta 1,alb}$	pdist1Alb	Fixed		0.2
Proportion of population in pulses all year	$p_{base,alb}$	pbaseAlb	Fixed		0.15
Minimum mortality (at $Temp_c \pm W$)	$\mu_{min,alb}$	mu22Alb	Fixed		0.06
Temperature – mortality slope	$\mu_{sl,alb}$	muslAlb	Uni	0.05-0.15	
Virus development at 22.5°C	$\gamma_{22,alb}$	gamma22Alb	Fixed		0.25
Temperature – virus development slope	$\gamma_{sl,alb}$	gammaslAlb	Tri	0.004-0.02	0.015
Maximum recruitment	$\rho_{max,alb}$	maxrhoAlb	Tri	100-20000	5000
Total recruitment through year	$R_{tot,alb}$		calculated		
Aedes aegypti					
Transmission mosquito to human host	b_{aeg}	bHAeg	Tri	0.1-0.7	0.4
Transmission human host to mosquito	β_{aeg}	betaHAeg	Tri	0.1-0.7	0.4
Days between blood feeding on humans	α_{aeg}	alphaprimeAeg	Tri	1-5	3
Proportion of population in peak one	$p_{\delta 1,aeg}$	pdist1Aeg	Fixed		0.25
Proportion of population in pulses all year	$p_{base,aeg}$	pbaseAeg	Fixed		0.13
Minimum mortality (at $Temp_c \pm W$)	$\mu_{min,aeg}$	mu22Aeg	Fixed		0.1
Slope of Temperature – mortality line	$\mu_{sl,aeg}$	muslAeg	Uni	0.05-0.15	
Virus development at 22.5°C Virus development	$\gamma_{22,aeg}$	gamma22Aeg	Fixed		0.25
Temperature – virus development slope	$\gamma_{sl,aeg}$	gammaslAeg	Tri	0.004-0.02	0.015
Maximum recruitment	$\rho_{max,aeg}$	maxrhoAeg	Tri	100-20000	5000
Total recruitment through year	$R_{tot,aeg}$		calculated		

```
function CHIKV2sim(numruns)
%Usage: CHIKV2sim(numruns)
%This is the top level script for iterative CHIKV2 simulations.
%numruns = the number of simulations to run.
%CHIKV2runmodel.m is called iteratively with its runnum input
 parameter
%set from 1 to numruns.
%Simulation progress is logged to CHIKV2simlog.txt.
%Simulation solutions are saved as matlab formatted files.
close all;       %close any open figures
if nargin < 1, numruns = 250; end %default numruns value
scrsz = get(0,'ScreenSize');
logfid = fopen('CHIKV2simlog.txt','wt');

fprintf(logfid,'Start simulations: %s\n', datestr(now));

%initialize results array
maxHi = NaN(numruns,2);   %Maximum human infection and location

for runnum = 1:numruns
 tic;
 CHIKV2sol = CHIKV2runmodel(runnum);
 fprintf(logfid,...
  'Run%3u simulation complete in %g seconds\n',runnum, toc);
  spoints = linspace(0,730,7301);

 [sy,syp] = deval(CHIKV2sol,spoints);
 spt  = spoints';   %transposed to meet column limits
 syt  = sy';      %when written to text or excel files
 sypt = syp';

 textarray = [spt syt sypt]; %build a text array for output

 [maxHi(runnum,1),maxHi(runnum,2)] = max(syt(:,2));
 namestring = ['Run' num2str(runnum)];
 close all;     %close any open figures

 %save solution
 save(namestring,'CHIKV2sol','spt','syt','sypt');

end
save('maxHi','maxHi');    %save maximum human infection array
fprintf(logfid,'End simulations: %s\n', datestr(now));
fclose('all');

figure
hist(maxHi(:,1),20)
xlabel('Maximum Human Infection');
ylabel('Count');
title('Maximum Human Infection Histogram');
print ('-dmeta', '-r1200','MaxHihist');
saveas(gcf, 'MaxHihist', 'fig');
```

```
end %CHIKV2sim
```

Published with MATLAB® R2019b

```
function [sol,tcrit] = CHIKV2runmodel(runnum)

%
%This is the Chikungunya virus (CHIKV2) model.
%Variants of the model have threshold (Temp_c) parameters hard coded
%in parameter list below, at 10, 16 or 22.

%model code published with Matlab 2019b.  Programmed and simulations
 run
% with Matlab versions 2015-2018.



if nargin < 1, runnum = 250; end %default runnum value
%runnum = 34;      %to run single parameter sets

daylim = 730;    %simulation end day

%load simulation parameters from file
%see table at start to map parameter names here to equations
load('./../CHIKV2paramgen/CHIKV2params.mat','params')

%assign params to named variables
Tmean  = params(runnum,1);  %mean of temperature curve
%human host
recH  = params(runnum,2);  %recovery rate
initH  = params(runnum,3);  %initial number of humans
mH   = params(runnum,4);  %mortality
baseH  = params(runnum,5);  %recruitment
tcrit  = params(runnum,6);  %day virus is introduced
%Mosquito Population Aedes albopictus (Alb)
bHAlb  = params(runnum,7);  %transmission to human host
betaHAlb = params(runnum,8);
alphaprimeAlb = params(runnum,9); %days between meals
intervalAlb = params(runnum,10); %interval between pulses
pdist1Alb = params(runnum,11); %proportion of population in peak one
pbaseAlb = params(runnum,12); %proportion of population in pulses all
 year
mean1Alb = params(runnum,13); %mean day peak one(from January 1)
sigma1Alb = params(runnum,14); %spread peak one
mean2Alb = params(runnum,15); %mean day peak two(from January 1)
sigma2Alb = params(runnum,16); %spread peak two
mu22Alb  = params(runnum,17); %mortality at temp=muthresh, also
 minimum mortality
muslAlb  = params(runnum,18); %slope of Temp-mortality
gamma22Alb = params(runnum,19); %virus devel at 22.5
gammaslAlb = params(runnum,20); %slope of virus devel
maxrhoAlb = params(runnum,21); %total Alb recruitment through year
%Mosquito Population Aedes aegypti (Aeg)
bHAeg  = params(runnum,22); %transmission to human host
betaHAeg = params(runnum,23);
alphaprimeAeg = params(runnum,24); %days between meals
```

```
intervalAeg = params(runnum,25); %interval between pulses
pdist1Aeg = params(runnum,26); %proportion of population in peak one
pbaseAeg = params(runnum,27); %proportion of population in pulses all
 year
mean1Aeg = params(runnum,28); %mean day peak one(from January 1)
sigma1Aeg = params(runnum,29); %spread peak one
mean2Aeg = params(runnum,30); %mean day peak two(from January 1)
sigma2Aeg = params(runnum,31); %spread peak two
mu22Aeg  = params(runnum,32); %vector mortality
muslAeg  = params(runnum,33); %slope of Temp-mortality
gamma22Aeg = params(runnum,34); %virus development
gammaslAeg = params(runnum,35); %slope of virus devel
maxrhoAeg = params(runnum,36); %total Aeg recruitment through year


%fixed parameters
meanH  = 99;  %time of peak recruitment
      %days from January 1 start of simulation
sigmaH  = 99;  %spread around meanH
incrH  = 0;
pHmeanAlb = 1;  %mean of human bias function
pHamplAlb = 0;  %amplitude of human bias function
offsetAlb = 0;  %offset for human bias
pHmeanAeg = 1;  %mean of human bias function
pHamplAeg = 0;  %amplitude of human bias function
offsetAeg = 0;  %offset for human bias
muthreshAlb = 10;  %temp threshold (+2-2) for minimum mortality
gammathreshAlb = 10; %lower temp threshold for virus development
muthreshAeg = 10;  %temp threshold (+2-2) for minimum mortality
gammathreshAeg = 10; %lower temp threshold for virus development

%derived parameter calculations
peakH = incrH * initH;
numH = initH + peakH;
totalAlb = maxrhoAlb * numH;
totalAeg = maxrhoAeg * numH;
NoOfPulseAlb = 365/intervalAlb;      %per year basis
pulseAlb = round((pbaseAlb * totalAlb)/(NoOfPulseAlb+1));
periodAlb = 2*pi/intervalAlb;
testtAlb = (intervalAlb-0.5)+(intervalAlb/2);
thresholdAlb = -cos(periodAlb*testtAlb);
NoOfPulseAeg = 365/intervalAeg;      %per year basis
pulseAeg = round((pbaseAeg * totalAeg)/(NoOfPulseAeg+1));
periodAeg = 2*pi/intervalAeg;
testtAeg = (intervalAeg-0.5)+(intervalAeg/2);
thresholdAeg = -cos(periodAeg*testtAeg);

%load temperature data from file
load('./../weatherdata/FloridaNormals.mat',...
 'FloridaMaxAvg','FloridaMinAvg','FloridaMaxRange','FloridaMinRange')

%function to convert Fahrenheit to Celsius
F2C = @(x)(x - 32) * 5/9;
```

```
%convert the florida normals temperature data to Celsius
hmean = F2C(FloridaMaxAvg);   %converts a temperature
lmean = F2C(FloridaMinAvg);
hrange = FloridaMaxRange * 5/9;     %converts a temperature range
lrange = FloridaMinRange * 5/9;

%calculate the temperature range from the Tmean parameter
Trange = lrange + (hrange-lrange)*(hmean-Tmean)/(hmean-lmean);

%y0 is a column vector for the initial values of the 15 state
 variables
 %human host
y0 = [initH   %suseptible
  0   %infected
  0   %recovered
 %Mosquito Population Alb
 pulseAlb  %suseptible
  0   %latent
  0   %infectious
 %Mosquito Population Aeg
 pulseAeg  %suseptible
  0   %latent
  0   %infectious

  0   %Alb recruitment accum, for output
  0   %Aeg recruitment accum, for output
  0   %human recruitment accumulator, for output
  0   %infected H accum, for output
  0   %infected Alb accum, for output
  0];   %infected Aeg accum, for output

%ode45 built-in defaults: 'RelTol',1e-3,'AbsTol',1e-6

options = odeset('MaxStep',1,'RelTol',1e-6,'Events',@P2events,...
 'NonNegative',[],'Refine',1);

sol = ode45(@gradient,[0 daylim],y0,options);

%The solution is expected to terminate at tcrit (prior to 365).
%It is then re-started with one infected human.
    % ynew is a column vector for the 15 new initial state variables
    ynew = sol.y(:,end);        %the last value of the 15 states
    ynew(2) = 1;                %one infected in Hi

%RelTol reduced to 1e-9 or smaller for best results
options = odeset('MaxStep',1,'RelTol',1e-9,'Events',@P2events,...
 'NonNegative',[],'Refine',1);

solnew = ode45(@gradient,[sol.x(end) daylim],ynew,options);
sol.x  = [sol.x  solnew.x(2:end)];  %skip first point (same as last
 point)
sol.y  = [sol.y  solnew.y(:,2:end)];
sol.xe = [sol.xe solnew.xe];
sol.ye = [sol.ye solnew.ye];
```

```
sol.ie = [sol.ie solnew.ie];
sol.stats.nsteps = sol.stats.nsteps + solnew.stats.nsteps;
sol.stats.nfailed = sol.stats.nfailed + solnew.stats.nfailed;
sol.stats.nfevals = sol.stats.nfevals + solnew.stats.nfevals;
sol.idata.f3d = cat(3,sol.idata.f3d,solnew.idata.f3d(:,:,2:end));

%The solution may terminate if the virus does not persist.
%The infected classes are checked for values less than 0.5. If this
 event
%is detected, the simulation is terminated and restarted with the
 infected
%classes set to zero values.
while sol.x(end) < daylim
 %at this point, there has been a terminal event and we are not
 finished
 % ynew is a column vector for the 15 new initial state variables
    ynew = sol.y(:,end);        %the last value of the 15 states
 ynew(2) = 0;    %zero Hi
 ynew(5) = 0;    %zero LAlb
 ynew(6) = 0;    %zero YAlb
 ynew(8) = 0;    %zero LAeg
 ynew(9) = 0;    %zero YAeg

 solnew = ode45(@gradient,[sol.x(end) daylim],ynew,options);
 sol.x  = [sol.x  solnew.x(2:end)];  %skip first point (same as last
 point)
 sol.y  = [sol.y  solnew.y(:,2:end)];
 sol.xe = [sol.xe solnew.xe];
 sol.ye = [sol.ye solnew.ye];
 sol.ie = [sol.ie solnew.ie];
 sol.stats.nsteps = sol.stats.nsteps + solnew.stats.nsteps;
 sol.stats.nfailed = sol.stats.nfailed + solnew.stats.nfailed;
 sol.stats.nfevals = sol.stats.nfevals + solnew.stats.nfevals;
 sol.idata.f3d = cat(3,sol.idata.f3d,solnew.idata.f3d(:,:,2:end));
end %while
%toc


%-----------------------------------------------------------------------
%Nested functions - allows base variables to be visible within
 functions
%
function dydt = gradient(time,state)
%assign some state variables to named variables for convenience
%human host
   Hs   = state(1);    %suseptible
   Hi = state(2);    %infected
   Hr = state(3);    %recovered
%Mosquito Population Alb
   SusAlb  = state(4);   %suseptible
   LAlb    = state(5);   %latent
   YAlb    = state(6);   %infectious
%Mosquito Population Aeg
   SusAeg  = state(7);   %suseptible
```

```
    LAeg     = state(8);   %latent
    YAeg     = state(9);   %infectious

 allH = Hs+Hi+Hr;

  %temperature model
  %
  %time is adjusted by -26.5 days to align the temperature model
  maximum
  %to the predominant maximum from the temperature data
  %(day 209, based on median(FloridaMaxsIdx) = 209)
  %2*pi/365 converts adjusted time in days to radians, 0 to 2*pi
  %The 0-1 day for simulation is January 1.
  %maximum summer temperature (when -cos((time-26.5)*2*pi/365) = 1),
  %is when (time-26.5)*2*pi/365 = pi; time = 209
  Temp = (Trange/2)*(-cos((time-26.5)*2*pi/365)) + Tmean;

  %define some functions of temperature
  %minimum mu = mu22, maximum mu = 1
     if Temp < muthreshAlb
         muAlb = min(1, max(mu22Alb,-muslAlb * (Temp - muthreshAlb + 2)
  + mu22Alb));
     else
         muAlb = min(1, max(mu22Alb, muslAlb * (Temp - muthreshAlb - 2)
  + mu22Alb));
     end
     if Temp < muthreshAeg
         muAeg = min(1, max(mu22Aeg,-muslAeg * (Temp - muthreshAeg + 2)
  + mu22Aeg));
     else
         muAeg = min(1, max(mu22Aeg, muslAeg * (Temp - muthreshAeg - 2)
  + mu22Aeg));
     end
  %gamma = gammasl * (Temp -22.5) + gamma22;
  if Temp < gammathreshAlb
   gammaAlb = 0;
  else
   gammaAlb = max(0,gammaslAlb*(Temp-22.5) + gamma22Alb); %protect from
  neg
  end
  if Temp < gammathreshAeg
   gammaAeg = 0;
  else
   gammaAeg = max(0,gammaslAeg*(Temp-22.5) + gamma22Aeg); %protect from
  neg
  end

  %susceptible vectorAlb,Aeg recruitment
 %  diff1Alb= mean1Alb-time;
 %  diff2Alb= mean2Alb-time;
   diff1Alb= min([abs(mean1Alb-time),abs(mean1Alb+365-time),...
   abs(mean1Alb-365-time),abs(mean1Alb+730-time)]);
   diff2Alb= min([abs(mean2Alb-time),abs(mean2Alb+365-time),...
   abs(mean2Alb-365-time),abs(mean2Alb+730-time)]);
```

```
  temp1Alb= sigma1Alb*sqrt(2*pi);
  temp2Alb= sigma2Alb*sqrt(2*pi);
  dist1Alb= (1/(temp1Alb)) * exp(-(diff1Alb^2)/(2*(sigma1Alb^2)));
  dist2Alb= (1/(temp2Alb)) * exp(-(diff2Alb^2)/(2*(sigma2Alb^2)));
  pdist2Alb= 1- (pbaseAlb + pdist1Alb);
%  diff1Aeg= mean1Aeg-time;
%  diff2Aeg= mean2Aeg-time;
  diff1Aeg= min([abs(mean1Aeg-time),abs(mean1Aeg+365-time),...
  abs(mean1Aeg-365-time),abs(mean1Aeg+730-time)]);
  diff2Aeg= min([abs(mean2Aeg-time),abs(mean2Aeg+365-time),...
  abs(mean2Aeg-365-time),abs(mean2Aeg+730-time)]);
  temp1Aeg= sigma1Aeg*sqrt(2*pi);
  temp2Aeg= sigma2Aeg*sqrt(2*pi);
  dist1Aeg= (1/(temp1Aeg)) * exp(-(diff1Aeg^2)/(2*(sigma1Aeg^2)));
  dist2Aeg= (1/(temp2Aeg)) * exp(-(diff2Aeg^2)/(2*(sigma2Aeg^2)));
  pdist2Aeg= 1- (pbaseAeg + pdist1Aeg);

%This treatment seems sufficient, ode45 handles the discontinuties OK.
  testrhoAlb = pdist1Alb*totalAlb*dist1Alb +
 pdist2Alb*totalAlb*dist2Alb;
  periodAlb  = (2*pi) /intervalAlb;
  testrhoAeg = pdist1Aeg*totalAeg*dist1Aeg +
 pdist2Aeg*totalAeg*dist2Aeg;
  periodAeg  = (2*pi) /intervalAeg;
  test_value = [testrhoAlb-1
            thresholdAlb+cos(periodAlb*(time+(intervalAlb/2)))
            testrhoAeg-1
            thresholdAeg+cos(periodAeg*(time+(intervalAeg/2)))];

% Mosquito Population Alb
  if (test_value(1) >= 0)       %distributed recruit >1
      if (test_value(2) < 0)        %and time for pulse
          rhoAlb = pulseAlb + testrhoAlb;
      else                          %and not time for pulse
          rhoAlb =          testrhoAlb;
      end
  else                          %distributed recruit <1
      if (test_value(2) < 0)        %and time for pulse
          rhoAlb = pulseAlb;
      else                          %and not time for pulse
          rhoAlb = 0;
      end
  end
% Mosquito Population Aeg
  if (test_value(3) >= 0)       %distributed recruit >1
      if (test_value(4) < 0)        %and time for pulse
          rhoAeg = pulseAeg + testrhoAeg;
      else                          %and not time for pulse
          rhoAeg =          testrhoAeg;
      end
  else                          %distributed recruit <1
      if (test_value(4) < 0)        %and time for pulse
          rhoAeg = pulseAeg;
      else                          %and not time for pulse
```

```
            rhoAeg = 0;
        end
    end

    %human recruitment (disabled by fixed parameter choices)
    temp3= peakH/(sigmaH*sqrt(2*pi));
%   diff3= meanH-time;
    diff3= min([abs(meanH-time),abs(meanH+365-time),abs(meanH+730-
time)]);
    RecruitH = baseH + temp3*exp(-(diff3^2)/(2*(sigmaH^2)));

%biased biting rates (kept for future use, here disabled by fixed
 parameter choices)
%cos goes from -1 to +1, check the amplitude range of result
% Mosquito Population Alb
pHAlb = pHmeanAlb + pHamplAlb*cos((time-offsetAlb)*2*pi/365); %prop
 feeding on humans
alphaAlb = alphaprimeAlb/pHAlb;                         %days/meal on
 humans
aHAlb = 1/alphaAlb;
% Mosquito Population Aeg
pHAeg = pHmeanAeg + pHamplAeg*cos((time-offsetAeg)*2*pi/365); %prop
 feeding on humans
alphaAeg = alphaprimeAeg/pHAeg;                         %days/meal on
 humans
aHAeg = 1/alphaAeg;

%EQUATIONS
%calculate terms for the derivative equations
dHs2i = ((aHAlb*bHAlb)/allH)*Hs*YAlb + ((aHAeg*bHAeg)/allH)*Hs*YAeg;
dHs   = RecruitH - (mH*Hs) - dHs2i;
dHi = dHs2i - (recH*Hi) - mH*Hi;
dHr = recH*Hi - mH*Hr;
%{
Currently mH=RecruitH=0; params kept as placeholders for later use
%}

dSusAlb = rhoAlb-(muAlb*SusAlb)-(aHAlb*betaHAlb*(Hi/allH))*SusAlb;
dLAlb = (aHAlb*betaHAlb*(Hi/allH))*SusAlb -muAlb*LAlb-gammaAlb*LAlb;
dYAlb = gammaAlb*LAlb-muAlb*YAlb;

dSusAeg = rhoAeg-(muAeg*SusAeg)-(aHAeg*betaHAeg*(Hi/allH))*SusAeg;
dLAeg = (aHAeg*betaHAeg*(Hi/allH))*SusAeg -muAeg*LAeg-gammaAeg*LAeg;
dYAeg = gammaAeg*LAeg-muAeg*YAeg;

    %dydt is a column vector for the "gradient" of the 19 state
 variables
    %human host
dydt = [dHs      %[1]susceptible
        dHi      %[2]infected
        dHr      %[3]recovered
    %vector Alb
        dSusAlb     %[4]susceptible
        dLAlb     %[5]latent
```

```
        dYAlb     %[6]infectious
  %vector Aeg
        dSusAeg     %[7]susceptible
        dLAeg     %[8]latent
        dYAeg     %[9]infectious
        rhoAlb     %[10]vector Alb recruitment accum
        rhoAeg     %[11]vector Aeg recruitment accum
        RecruitH    %[12]human recruitment accum
        dHs2i     %[13]infectious H accum
        gammaAlb*LAlb   %[14]infectious Alb accum
        gammaAeg*LAeg];   %[15]infectious Aeg accum
end %gradient

%This format for the events function is required by the ode45
 function.
function [value,isterminal,direction] = P2events(time,y)
 Hitest   = y(2) < 0.5;
 LAlbtest = y(5) < 0.5;
 LAegtest = y(8) < 0.5;
 YAlbtest = y(6) < 0.5;
 YAegtest = y(9) < 0.5;
 alltest = Hitest&LAlbtest&LAegtest&YAlbtest&YAegtest;

    value = [time-tcrit
    alltest-0.5];
 if time <= tcrit
   isterminal = [1; 0];
 else
   isterminal = [1; 1];
 end %if
    direction  = [1; 1];
end


end
```

Published with MATLAB® R2019b
